# Estimating the dietary and health impact of implementing front-of-pack nutrition labeling in Canada: A macrosimulation modeling study

**DOI:** 10.3389/fnut.2023.1098231

**Published:** 2023-03-17

**Authors:** Nadia Flexner, Alena P. Ng, Mavra Ahmed, Neha Khandpur, Rachel B. Acton, Jennifer J. Lee, Mary R. L’Abbe

**Affiliations:** ^1^Department of Nutritional Sciences, Temerty Faculty of Medicine, University of Toronto, Toronto, ON, Canada; ^2^Joannah and Brian Lawson Centre for Child Nutrition, Temerty Faculty of Medicine, University of Toronto, Toronto, ON, Canada; ^3^Department of Human Nutrition and Health, Wageningen University, Wageningen, Netherlands; ^4^Center for Epidemiological Research in Nutrition and Health, School of Public Health, University of São Paulo, São Paulo, Brazil; ^5^Department of Nutrition, Harvard T. H. Chan School of Public Health, Harvard University, Boston, MA, United States; ^6^School of Public Health and Health Systems, University of Waterloo, Waterloo, ON, Canada

**Keywords:** front-of-pack nutrition label, dietary intakes, diet-related NCD, NCD and risk factors, macrosimulation model, food policy

## Abstract

**Background:**

Front-of-pack labeling (FOPL) has been identified as a cost-effective policy to promote healthy diets. Health Canada has recently published FOPL regulations that will require food and beverages that meet or exceed set thresholds for sodium, sugars, or saturated fat to display a ‘high in’ symbol on the front of the package. Although a promising measure, its potential impact on dietary intakes and health have not yet been estimated in Canada.

**Objective:**

This study aims to estimate (1) the potential dietary impact of implementing a mandatory FOPL among Canadian adults; and (2) the number of diet-related non-communicable disease (NCD) deaths that could be averted or delayed due to these estimated dietary changes.

**Methods:**

Baseline and counterfactual usual intakes of sodium, total sugars, saturated fats, and calories were estimated among Canadian adults (*n* = 11,992) using both available days of 24 h recalls from the 2015 Canadian Community Health Survey-Nutrition. The National Cancer Institute method was used to estimate usual intakes, and adjusted for age, sex, misreporting status, weekend/weekday, and sequence of recall. Estimated counterfactual dietary intakes were modeled from reductions observed in experimental and observational studies that examined changes in sodium, sugars, saturated fat, and calorie content of food purchases in the presence of a ‘high in’ FOPL (four counterfactual scenarios). The Preventable Risk Integrated ModEl was used to estimate potential health impacts.

**Results:**

Estimated mean dietary reductions were between 31 and 212 mg/day of sodium, 2.3 and 8.7 g/day of total sugars, 0.8 and 3.7 g/day of saturated fats, and 16 and 59 kcal/day of calories. Between 2,183 (95% UI 2,008–2,361) and 8,907 (95% UI 8,095–9,667) deaths due to diet-related NCDs, mostly from cardiovascular diseases (~70%), could potentially be averted or delayed by implementing a ‘high in’ FOPL in Canada. This estimation represents between 2.4 and 9.6% of the total number of diet-related NCD deaths in Canada.

**Conclusion:**

Results suggest that implementing a FOPL could significantly reduce sodium, total sugar, and saturated fat intakes among Canadian adults and subsequently prevent or postpone a substantial number of diet-related NCD deaths in Canada. These results provide critical evidence to inform policy decisions related to implementing FOPL in Canada.

## 1. Introduction

In Canada, more than 80% of all deaths are due to non-communicable diseases (NCDs) (1). Canadians’ intakes of nutrients of public health concern (i.e., saturated fats, sodium and sugars) are above recommended levels to prevent diet-related NCDs (2–7). It is well established that unhealthy diets are one of the major preventable risk factors for a range of NCDs (8). Diets are influenced by many factors and one of them is the food environment, which plays a key role in consumer food choices (9). The government of Canada has taken several actions to improve the Canadian food environment (10). In 2016, as part of the *Healthy Eating Strategy*, Health Canada proposed the implementation of front-of-pack labeling (FOPL) to provide consumers with simple and easy to understand nutrition labeling (10). Accordingly, Health Canada has recently published FOPL regulations in *Canada Gazette II (July, 2022)*, requiring prepackaged foods and beverages that meet or exceed set thresholds for saturated fats, sodium, and sugars to display a mandatory ‘high in’ nutrition symbol on the front of the package, to come into effect by January 2026 (11, 12).

Front-of-pack labeling has been recognized by international public health organizations as a policy priority for promoting healthy diets, as it allows consumers at different levels of literacy to make informed decisions about the nutritional quality of foods they purchase and consume and promotes healthier food environments (13–18). Adoption of FOPL is one of the World Health Organization’s (WHO) ‘Best Buys’ most cost-effective recommendations for the prevention and control of diet-related NCDs (19, 20).

The purpose of a FOPL system is to assess the nutritional quality of a food or beverage product and display it in a simple, easy to interpret visual form to promote healthy dietary behaviors; and to motivate the food industry to reformulate food products (14, 21–31). For the most part, FOPL systems can be classified into two major types: interpretive and non-interpretive. Interpretive labels provide nutrition information on individual nutrients and provide an interpretation or recommendation for the consumer (e.g., traffic light label, ‘high in’ symbols, star-based systems, and Nutriscore). Non-interpretive labels, on the other hand, provide only information on nutrients, but without any interpretation [e.g., % Guideline Daily Amount (GDA) system] (14).

Although there is currently no consensus yet on which FOPL scheme performs better, increasing evidence demonstrates that the ‘high in’ FOPL system (also known as nutrient warning) has the potential to provide clear and easy to understand information that allows consumers to quickly identify food and beverage products that have high levels of critical nutrients (i.e., saturated fats, sodium, and sugars) linked to several risk factors and diet-related NCDs (29, 32, 33). For instance, ‘high in’ FOPL symbols have been shown to help consumers make healthier food choices (28, 34–39), and improve their understanding of excess nutrient content and their ability to identify healthier food products (22, 28, 32, 34, 40). A recent meta-analysis showed that nutrient warning labels, such as the ‘high in’ symbol, performed better than other FOPL systems at discouraging unhealthful consumer purchasing behavior, and significantly decreased the calorie and total saturated fat content of food and beverage purchases (41).

In 2016, Chile was the first country to adopt a ‘high in’ FOPL system as part of the comprehensive Chilean Food Labeling and Marketing Law – Law 20.606 (42). The Law requires processed food products that exceed established limits for calories and certain critical nutrients (i.e., saturated fats, sodium, and sugars) to be labeled with a ‘high in’ symbol. Previous, early evaluations of this Law showed reductions of nearly 24% in sugar sweetened beverage (SSBs) purchases (43); small but significant declines in the calorie, sodium, saturated fat, and sugar content of households’ food and beverage purchases (44); and industry driven food reformulation (45–48). Furthermore, evaluations of this policy suggest that consumers shifted consumption toward products displaying no ‘high in’ labels and that these effects persisted overtime, and most of the decrease in purchases of ‘high in’ food products was compensated for by an increase in purchase of products displaying no ‘high in’ labels (44, 47) – the net effect was an overall reduction in nutrients of public health concern (44).

More recently, ‘high in’ or ‘excess’ FOPL approaches have been adopted and/or implemented in Peru (49), Uruguay (50), Israel (51), Mexico (52), Argentina (53), Colombia (54), Brazil (55), Venezuela (56), and Canada (11, 12). However, the long term potential impacts of FOPL have yet to be determined, given how nascent these policies are. Therefore, in order to better understand the long term impacts of implementing a FOPL system, simulation modeling methods can be used to estimate the potential impacts of this policy on dietary and health outcomes. Simulation modeling methods have proven to be an effective tool in the policymaking process, as they estimate the impact of initiatives before actual policy implementation (57, 58). Using the best available evidence, impact is estimated using a baseline scenario and counterfactual scenarios for the proposed policy – in this case the potential impact of implementing a ‘high in’ FOPL symbol in Canada. There have been a number of studies supporting implementation of FOPL in Canada (40, 59, 60); however, to our knowledge, population-level dietary and health impacts of implementing a ‘high in’ FOPL symbol in Canada have not been previously estimated.

Therefore, the objectives of the current study are to estimate (1) the potential dietary impact among Canadian adults of implementing a mandatory ‘high in’ FOPL based on actual experimental and observational evidence; and (2) the number of diet-related NCD deaths that could be averted or delayed due to these estimated dietary changes using simulation modeling methods.

## 2. Materials and methods

### 2.1. Baseline dietary data: CCHS - Nutrition 2015

The *Canadian Community Health Survey (CCHS)-Nutrition 2015* is a nationally representative, cross-sectional health and nutrition survey conducted by Statistics Canada, which uses 24 h dietary recalls to collect data on food and beverage intake in Canada. Briefly, *CCHS-Nutrition 2015* was conducted *via* computer-assisted in-person interviews by Statistics Canada trained personnel, collecting data on 20,487 individuals. The first 24 h recall was conducted during the in-person interviews. The second 24 h recall was conducted *via* telephone on 35% of the sample within 3 to 10 days after the first recall (61). For dietary recalls, *CCHS-Nutrition 2015* used a Canadian modification of the United States Department of Agriculture (USDA) 5-step Automated Multiple-Pass method, because of its known strength in capturing dietary intakes with less misreporting bias (62). Respondents included those aged ≥1 year residing in private dwellings in 10 provinces of Canada and excluded those residing in the territories, on reserves and other indigenous settlements, in some remote areas or in institutions (e.g., care facilities or prisons), or individuals who were full-time members of the Canadian Armed Forces (61).

For this study, both available days of 24 h dietary recalls from the publicly available *CCHS-Nutrition 2015 Public Use Microdata File (PUMF)* (61, 63) were used to estimate usual dietary intakes by Dietary Reference Intakes (DRI) (64) age-sex groups for sodium, total sugars, saturated fats, and calories, which was used as the baseline scenario. To obtain energy and nutrient content of all foods reported in *CCHS-Nutrition 2015*, we used information from Health Canada’s Canadian Nutrient File (CNF), version 2015 (65). The CNF provides food composition data for up to 152 food components for 5,690 food products commonly consumed in Canada (i.e., fresh, packaged, and prepared foods and beverages) (65).

In this analysis we included Canadian adults (≥19 years; *n* = 13,919) and excluded breastfeeding women (n = 188) and respondents who did not report any food consumption (*n* = 4). Additionally, underweight respondents with a BMI <18.5 kg/m^2^ were excluded, since energy expenditure equations for this population are not available, and those without self-reported or measured height and weight (*n* = 1,735). After exclusions, a total of 11,992 participants were included in this study (4). The energy intake to total energy expenditure ratio was calculated for each respondent in order to adjust for dietary misreporting, which has been detailed elsewhere by our research group (4).

### 2.2. FOPL counterfactual scenarios

For the FOPL counterfactual scenarios used in this study, the most recent available data from observational and experimental studies (40, 41, 44) on the potential reduction of critical nutrients (i.e., saturated fats, sodium, and sugars) and calorie content of food and beverage purchases in the presence of a ‘high in’ FOPL symbol was used. Scenarios 1 and 2 were based on the early evaluations of the Chilean Food Labeling and Marketing Law (44); scenario 3 was based on a Canadian randomized experimental marketplace study (40); and scenario 4 was based on results from a recent meta-analysis on the impact of nutrient warning labeling (41). All counterfactual scenarios captured changes in consumer purchasing behavior (e.g., food substitution, decrease in the amount of ‘high in’ foods consumed). In addition, scenarios 1 and 2 most likely also captured initial food reformulation.

**Figure 1:**
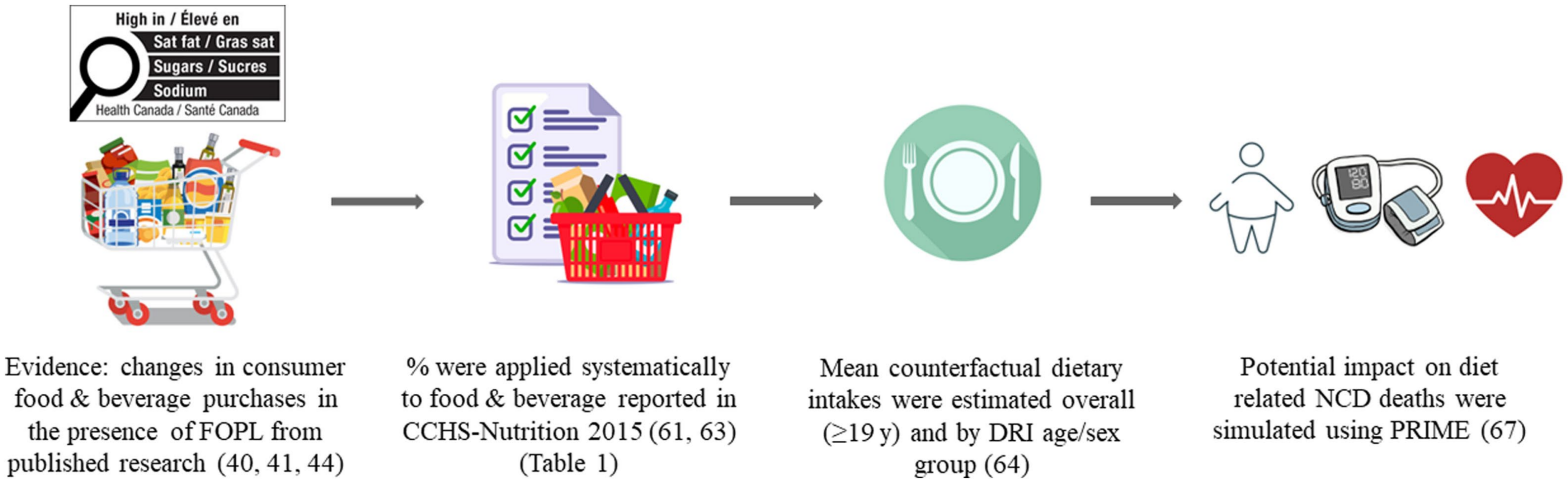
Overview of the steps followed in the simulation modeling study. Full details of each counterfactual scenario and sensitivity analysis are described in the methods section.

According to the reductions identified in each counterfactual scenario, changes in nutrient intakes were modeled by decreasing the nutrient composition of foods and beverages consumed by Canadian adults *(CCHS-Nutrition 2015)*, except for alcoholic beverages and meal replacements, which are regulated separately and are not part of the recently published Canadian ‘high in’ FOPL regulation. Then, ‘new’ usual dietary intakes were estimated for all adults and by DRI age-sex groups (Figure 1).

The four FOPL counterfactual scenarios and nutrient reductions applied in this study are detailed below and summarized in Table 1.

**Table 1:** Percentage change of critical nutrients and calorie content of food and beverage purchases applied for each FOPL counterfactual scenario (summary).

Counterfactual scenarios	Source	Tested foods	Main analysis			Sensitivity analysis
			∆Sodium	∆Sugars	∆Saturated fat	∆Calories
Scenario 1	Based on early FOPL evaluations from Chile (44)	Food and beverages overall	–4.7%	–10.2%	–3.9%	−3.5%
Scenario 2	Based on early FOPL evaluations from Chile (44)	Foods	−4.6%	−5.4%	−3.6%	−1.7%
		Beverages	−5.2%	−13.2%	−5.6%	−9.9%
Scenario 3	Based on a Canadian randomized experimental marketplace study (40)	Snack foods	−6.3%	−0.1%	−6.5%	−3.0%
		Beverages	−5.5%	−8.7%	−19.5%	−10.5%
Scenario 4	Based on results from a systematic review and meta-analysis (41)	Food & beverages overall	−7.8%	−7.3%	−16.3%	−12.9%

Full details of each counterfactual scenario and sensitivity analysis are described in the methods section.

#### 2.2.1. Scenario 1: Based on early evaluations of the Chilean Food Labeling and Marketing Law – overall changes

Scenario 1 (S1) was based on early evaluations of the Chilean Food Labeling and Marketing Law, examining changes in the calorie, sugar, sodium, and saturated fat content of food and beverage purchases following the first phase of implementation (44). The evaluation used longitudinal data (January 1, 2015, to December 31, 2017) on households’ food and beverage purchases captured before and after implementation of the Chilean Law (2,381 households) (44). Results showed small but significant declines in overall household purchases of calories (−3.5%), saturated fat (−3.9%), sodium (−4.7%), and sugars (−10.2%) (44). Overall reductions for calories, saturated fats, sodium and sugars were applied systematically to all foods and beverages reported in *CCHS-Nutrition 2015*, and then ‘new’ usual dietary intakes for all adults and by DRI age-sex groups were estimated.

#### 2.2.2. Scenario 2: Based on early evaluations of the Chilean Food Labeling and Marketing Law – changes disaggregated by food and beverages

Scenario 2 (S2) was also based on early evaluations of the Chilean Food Labeling and Marketing Law (44); however, in this scenario, reported changes were applied disaggregated by foods [calories (−1.7%), sugars (−5.4%), saturated fat (−3.6%), and sodium (−4.6%)], and beverages [calories (−9.9%), sugars (−13.2%), saturated fat (−5.6%), and sodium (−5.2%)] to compare versus applying changes across both foods and beverages as in S1. Reductions for calories, saturated fats, sodium and sugars were applied separately to foods and beverages reported by Canadian adults in *CCHS-Nutrition 2015*, and then ‘new’ usual dietary intakes for all adults and by DRI age-sex groups were estimated.

#### 2.2.3. Scenario 3: Based on a Canadian randomized experimental marketplace study – changes disaggregated by snack food and beverages

Scenario 3 (S3) was based on a Canadian randomized experimental marketplace study (40) that examined the relative impact of different FOPL systems on snack food and beverage purchases among individuals 13 years and older in Canada (*n* = 3,584). The study examined changes in the content of calories and key nutrients (i.e., saturated fats, sodium, and sugars) of snack food and beverage purchases. For the present study relative reductions observed in the presence of a ‘high in’ symbol versus no FOPL among participants 18 years and older were used, in order to be consistent with this study’s target population and to be conservative with our estimates given that relative changes were higher among participants <18 years. Observed relative reductions were reported separately for snack foods (calories (−3.0%), sugars (−0.1%), saturated fat (−6.5%), and sodium (−6.3%)), and beverages (calories (−10.5%), sugars (−8.7%), saturated fat (−19.5%), and sodium (−5.5%)). For this counterfactual scenario, all snack foods and beverages reported in *CCHS-Nutrition 2015* were identified using the Health Canada’s Table of References Amounts (TRA) food categories (see Supplementary Table S1). Reductions for calories, saturated fats, sodium and sugars were applied only to snack foods and beverages reported by Canadian adults in *CCHS-Nutrition 2015*, which makes this the most conservative counterfactual scenario modeled in this study. Reduced snack foods and beverage were then combined with the other foods reported by *CCHS-Nutrition 2015* respondents, and then ‘new’ usual dietary intakes for all adults and by DRI age-sex groups were estimated.

#### 2.2.4. Scenario 4: Based on a meta-analysis on the impact of nutrient warning labeling – overall changes

Scenario 4 (S4) was based on results from a recent meta-analysis of randomized controlled trials and quasi experimental studies that examined overall changes in the calorie and key nutrients (i.e., saturated fats, sodium, and sugars) content of food and beverage purchases in the presence of FOPL (41). Specifically, the results comparing a ‘high in’ FOPL symbol or warning label vs. control/no label or Nutrition Facts table (NFt) were used. Overall changes were applied for calories (−12.9%), sugars (−7.3%), saturated fat (−16.3%), and sodium (−7.8%) to all foods and beverages reported in *CCHS-Nutrition 2015*, and then ‘new’ usual dietary intakes for all adults and by DRI age-sex groups were estimated.

#### 2.2.5. Sensitivity analysis

For all scenarios tested in this study, changes in calorie intakes were estimated by calculating the calories contributed from changes in sugar and saturated fat [i.e., counterfactual kcals = baseline kcals – ((∆sugars (g) * 4 kcal) + (∆saturated fat (g) * 9 kcal))], in order to be conservative with our estimates. However, since evidence used to build these counterfactual scenarios (40, 41, 44) also provided estimates for changes in calories overall, sensitivity analyses were conducted using these proportions only (see Supplementary Table S3.1.1).

Additionally, an alternative scenario was modeled based on the criteria that WHO (20) used to estimate FOPL cost-effectiveness as part of the *Technical briefing for Appendix 3 of the Global Action Plan for NCDs* (20). This criterion is for FOPL systems overall and not specifically for ‘high in’ symbols. WHO also included fiber and trans fatty acids but did not include sugars in their criteria. Therefore, this scenario was included as a sensitivity analysis only. For this scenario, only reductions included in the proposed criteria for calories (−5.3%), saturated fat (−12.9%) and sodium (−6.4%) were applied.

### 2.3. Mean height and BMI data: CCHS - Nutrition 2015

For this study, mean height and body mass index (BMI) were calculated by DRI age-sex group for the same sample included in both the baseline and counterfactual scenario *(CCHS-Nutrition 2015)*. Previous research on both 2004 and 2015 cycles of *CCHS-Nutrition* has shown that adult women tend to under-report their weight, and adult men tend to over-report their height. To mitigate the introduction of these systematic biases into the analyses, BMI correction factors provided by Statistics Canada were used to generate respondents’ BMI and height from self-reported values, if measured values were not available (66). The final mean BMI and mean heights for the sample were generated from both corrected and measured values (Table 2). Mean height and BMI estimations were then used as inputs for simulation modeling.

**Table 2:** Sample characteristics, adults (≥19 year).

Sex, *n* (%)	All (*n* = 11,992)
Males	5,674 (50.1)
Females	6,318 (49.9)
Highest Level of Education, *n* (%)	
Less than high school diploma or its equivalent	1,938 (11.8)
High school diploma or a high school equivalency certificate	3,066 (25.0)
Certificate/diploma -trade/college/non-university/university below Bachelor	3,980 (34.2)
Bachelor’s degree or university certificate/diploma/degree above bachelor’s degree	2,937 (28.5)
Not stated	71 (0.6)
Total annual household income, all sources *n* (%)	
<$20,000	1,323 (7.9)
$20,000 – $39,999	2,520 (17.0)
$40,000 – $59,999	2,139 (16.5)
$60,000 – $79,999	1,679 (14.2)
$80,000 – $99,999	1,218 (11.1)
$100,000 – $119,999	992 (10.3)
$120,000 – $139,999	626 (6.9)
>$140,000	1,488 (16.0)
Not stated	7 (0.1)
Total household income, main source *n* (%)	
Employment income	7,709 (72.7)
Income from social benefits	396 (2.4)
Senior’s benefits	3,128 (19.0)
Other	422 (3.5)
Not stated	337 (2.4)
BMI, *n* (%)	
Normal weight (18.5–24.9 kg/m^2^)	3,920 (32.7)
Overweight (25.0–29.9 kg/m^2^)	4,409 (36.8)
Obese (>30.0 kg/m^2^)	3,663 (30.6)
Mean BMI, by DRI age-sex category (CI)	
19–30 (m)	26.14 (25.12, 27.16)
19–30 (f)	25.47 (24.67, 26.26)
31–50 (m)	28.50 (28.00, 28.98)
31–50 (f)	26.98 (26.52, 27.44)
51–70 (m)	29.05 (28.32, 29.79)
51–70 (f)	27.94 (27.33, 28.55)
71+ (m)	28.05 (27.53, 28.56)
71+ (f)	27.80 (27.03, 28.57)
Height (m), by DRI age-sex category (CI)	
19–30 (m)	1.77 (1.76, 1.77)
19–30 (f)	1.64 (1.63, 1.65)
31–50 (m)	1.76 (1.76, 1.77)
31–50 (f)	1.63 (1.62, 1.64)
51–70 (m)	1.74 (1.73, 1.75)
51–70 (f)	1.61 (1.60, 1.62)
71+ (m)	1.72 (1.71, 1.73)
71+ (f)	1.58 (1.57, 1.58)
Misreported, *n* (%)	
Under-reporter	4,567 (37.9)
Plausible	6,598 (55.3)
Over-reporter	827 (6.8)

### 2.4. Health impact modeling

The Preventable Risk Integrated ModEl (PRIME) (67), a cross-sectional NCD scenario model, was used to estimate the number of diet-related NCD deaths that could be averted or delayed due to potential dietary intake changes from implementing a ‘high in’ FOPL symbol in Canada (under the four counterfactual scenarios described above). PRIME is an open-access model developed by researchers at the University of Oxford (67) and endorsed by the WHO Regional Office for Europe (68). PRIME has been used extensively in many studies in different countries (69–88), including Canada (3, 89, 90).

PRIME includes alcohol consumption, smoking, physical activity, and diet (energy, fruits & vegetables, fiber, salt, total fat, saturated fat, unsaturated fat and cholesterol consumption) as behavioral risk factors and 24 different health outcomes (including cardiovascular diseases (CVDs), cancers, diabetes, kidney disease, chronic obstructive pulmonary disease, and liver disease) (67). Sugar is not included as a risk factor in PRIME, thus, its effect is mediated through calorie reduction. PRIME estimates the impact of population-level changes in the distribution of behavioral risk factors on population NCD mortality using evidence from robust meta-analysis from epidemiological studies, methods which have been previously described (67). The model, specifically, answers the question “How many deaths would have occurred in the baseline year if the distribution of risk factors had been different? (68).”

Inputs for the model included age- and sex-specific data for: (1) number of individuals in the population; (2) annual number of diet-related NCD deaths relevant to the study; (3) baseline population distribution of behavioral risk factors {Canadian adults’ calorie, sodium, and saturated fat [% of total energy (TE) intake at baseline]}; and (4) the counterfactual distribution of behavioral risk factors of interest (Canadian adults’ calorie, sodium, and saturated fat (%TE) intake after modeling reductions).

#### 2.4.1. Canadian specific population demographics and diet-related NCD mortality burden

Population demographics and mortality data (2019) associated with diet-related NCDs (CVDs, diabetes, cancer, chronic renal failure, and liver disease) were acquired from the publicly available Statistics Canada CANSIM tables (stratified by sex and 5-year age band) (91–96). The 2019 diet-related NCD mortality data was used, assuming no major changes in Canadians’ dietary intakes between 2015 (last *CCHS-Nutrition* survey) and 2019, and also because it is the most recent available data before the COVID-19 pandemic, which has disproportionally affected people with NCDs (97, 98) and has changed the way Canadians buy and eat foods (99–101) – impacts that remain to be evaluated. Diet-related NCD mortality data were based on the WHO International Classification of Diseases 10 (ICD 10) (102).

### 2.5. Statistical analyses

Both available 24 h recall days from *CCHS-Nutrition 2015 PUMF* were used to assess Canadian adults’ usual calorie and nutrient intakes (sodium, total sugars, saturated fat, and saturated fat (%TE)) for baseline (actual intakes) and counterfactual scenarios. The National Cancer Institute (NCI) method (103) was used to estimate usual intakes and distributions (overall and by DRI age-sex group), with adjustments for age, sex, dietary misreporting status, weekend/weekday, and sequence of dietary recall. The 1-part (amount only) model was used, as indicated by Davis et al. (104), because zero consumption of the studied nutrients was <5%. This method used stratified analysis by DRI age-sex groups and outlier removal for implausible nutrient intakes. The bootstrap balanced repeated replication method (500 replicates) was used for confidence intervals and standard error estimations.

To ensure nationally representative estimates, sample survey weights provided by Statistics Canada were applied to all analyses (61). Meaningfully differences between baseline (current intakes) and counterfactual dietary intakes were assessed using non-overlapping 95th percentile confidence limits of the means between baseline and counterfactual intakes, as in previous dietary pattern research (105, 106). SAS version 9.4 (SAS Institute Inc.) was used for analyses. SAS codes and data manipulation are available upon request from the authors.

For the scenario simulation modeling component, after inputting all the required data into PRIME, the model estimated changes in the number of deaths attributable to each diet-related NCD between the baseline and each of the counterfactual scenarios. In order to estimate 95% uncertainty intervals (UIs) around the results (based on 2.5^th^ and 97.5^th^ percentiles) Monte Carlo analysis built in PRIME was performed at 10,000 iterations, which allowed the epidemiological parameters to vary randomly according to the distributions considered in the model [published in the literature (67)]. PRIME estimated the number of diet-related NCD deaths that could have been averted or delayed for each of the counterfactual scenarios tested, overall and disaggregated by sex and each disease under study.

## 3. Results

### 3.1. Sample characteristics

Sample characteristics are shown in Table 2. Briefly, a total of 11,992 (≥19 years) *CCHS-Nutrition 2015* respondents were included in this analysis, 49.9% of them being females, around 87% reporting having at least high school diploma or high school equivalency certificate, and more than 44% reported having a household income greater than $80,000/year. Based on self-reported (adjusted) and measured BMI values, it was estimated that 32.7% of respondents could be classified as having normal-weight, 36.8% overweight and 30.6% obesity.

### 3.2. Baseline scenario: Actual usual calorie and nutrient intakes

**Figure 2:**
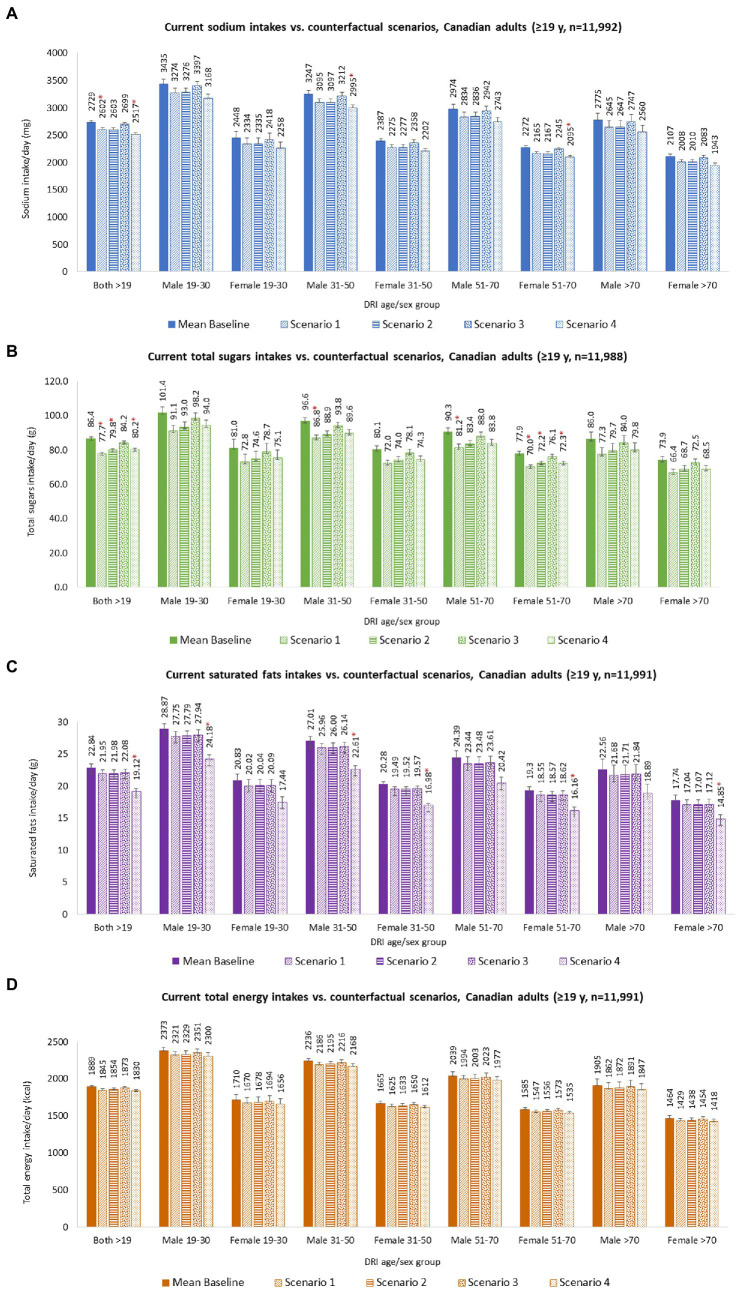
Nutrient intakes of Canadian adults (≥19 years), overall and by DRI age-sex groups, for baseline and under each counterfactual scenario. **(A)** Sodium intakes; **(B)** Total sugars intakes; **(C)** Saturated fats intakes; and **(D)** Total energy intakes. Baseline and counterfactual calorie and nutrient intakes were estimated using *CCHS-Nutrition 2015 PUMF data* (61, 63). Usual intakes were estimated using the National Cancer Institute (NCI) method (103), and analyses were adjusted for age, sex, dietary misreporting status, weekend/weekday, and sequence of dietary recall. d, day; g, grams; mg, milligrams; FIGURE 2 (Continued)kcal, kilocalories; SE, standard error. Counterfactual scenario 1 and 2 were based on Taillie et al. (44). SCENARIO 1, overall changes: sodium (mg) −4.7%; sugars (g) −10.2%; saturated fat (g) −3.9%; and SCENARIO 2, disaggregated by foods: sodium (mg) –4.6%; sugars (g) –5.4%; saturated fat (g) –3.6%; and beverages: sodium (mg) –5.2%; sugars (g) –13.2%; saturated fat (g) –5.6%. Counterfactual scenario 3 was based on Acton et al. (40). SCENARIO 3, relative changes disaggregated by snack foods: sodium (mg) –6.3%; sugars (g) –0.1%; saturated fat (g) –6.5%; and beverages: sodium (mg) –5.5%; sugars (g) –8.7%; saturated fat (g) –19.5%. Counterfactual scenario 4 was based on Song et al. (41). SCENARIO 4, overall changes: sodium (mg) –7.8%; sugars (g) –7.3%; saturated fat (g) –16.3%. * Indicates a statistically significant difference between baseline mean intakes and counterfactual mean intakes.

Calorie and nutrient intakes for Canadian adults overall and by DRI age-sex group are detailed in Supplementary Table S2. Canadian adults’ usual mean ± SE calorie and nutrient intakes were estimated as follows: calories 1889 ± 20 kcal/day, sodium 2,729 ± 33 mg/day, sugars 86.4 ± 0.9 g/day, saturated fat 22.8 ± 0.6 g/day, and saturated fat (%TE) 10.6 ± 0.18%/day (Figure 2 and Supplementary Table S2). Usual mean intakes disaggregated by food and beverage contribution were also estimated for calories (food 1,634 kcal/day; beverage 255 kcal/day), sodium (foods 2,572 mg/day; beverages 157 mg/day), sugars (food 59.9 g/day; beverage 27.4 g/day), saturated fat (food 21.1 g/day; beverage 2.0 g/day), and saturated fat (%TE) (food 11.17%/day; beverage 9.15%/day) (Supplementary Table S2).

### 3.3. FOPL counterfactual scenarios: Potential dietary and health gains

#### 3.3.1. S1: Based on early evaluations of the Chilean Food Labeling and Marketing Law – overall changes

Modeling S1 resulted in absolute mean dietary reductions of 128 mg/day sodium, 8.7 g/day sugars, 0.9 g/day saturated fats, and 43 kcal/day calories (considering only calorie contribution from changes observed in sugar and saturated fat) for adults overall (Figure 2 and Supplementary Table S3). Significant differences were observed for mean sodium and sugar intakes between baseline and counterfactual S1. Stratified results by DRI age-sex group were also estimated and are presented in Supplementary Table S3.

**Figure 3:**
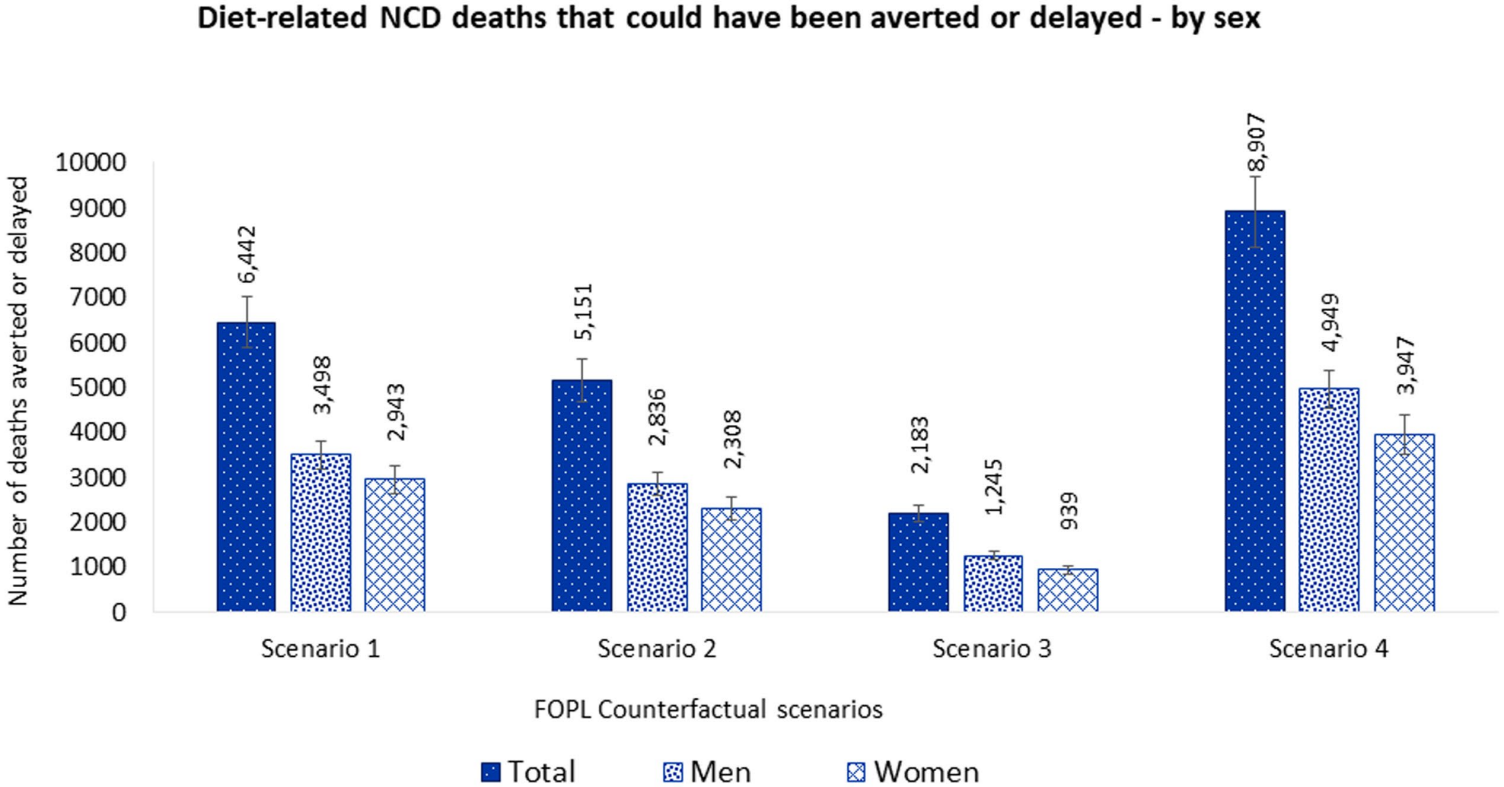
Number of diet-related NCD deaths that could be averted or delayed from implementing a ‘high in’ FOPL in Canada by sex. Potential diet-related NCD deaths that could be averted or delayed were estimated using the PRIME model (67). Inputs for the mode included, (1) population demographics; (2) mortality data associated with diet-related NCDs (CVDs, diabetes, cancer, chronic renal failure, and liver disease) (2019), obtained from the publicly available Statistics Canada CANSIM tables (stratified by sex and 5-year age band) (91–96); and (3) baseline and counterfactual dietary intakes estimations using *CCHS-Nutrition 2015 PUMF data* (61, 63). Counterfactual scenario 1 and 2 were based on Taillie et al. (44). SCENARIO 1, overall changes: sodium (mg) –4.7%; sugars (g) –10.2%; saturated fat (g) –3.9%; and SCENARIO 2, disaggregated by foods: sodium (mg) –4.6%; sugars (g) –5.4%; saturated fat (g) –3.6%; and beverages: sodium (mg) –5.2%; sugars (g) –13.2%; saturated fat (g) –5.6%. Counterfactual scenario 3 was based on Acton et al. (40). SCENARIO 3, relative changes disaggregated by snack foods: sodium (mg) –6.3%; sugars (g) –0.1%; saturated fat (g) –6.5%; and beverages: sodium (mg) –5.5%; sugars (g) –8.7%; saturated fat (g) –19.5%. Counterfactual scenario 4 was based on Song et al. (41). SCENARIO 4, overall changes: sodium (mg) –7.8%; sugars (g) –7.3%; saturated fat (g) –16.3%.

These estimated dietary intakes changes were estimated to prevent or postpone 6,442 (95% UI 5,870–7,020) deaths from diet-related NCDs. Approximately 54% of averted or delayed deaths were in men (3,498 [95% UI 3,193–3,802]) and 46% in women (2,943 [95% UI 2,618–3,259]) (Figure 3 and Supplementary Table S4.1).

**Figure 4:**
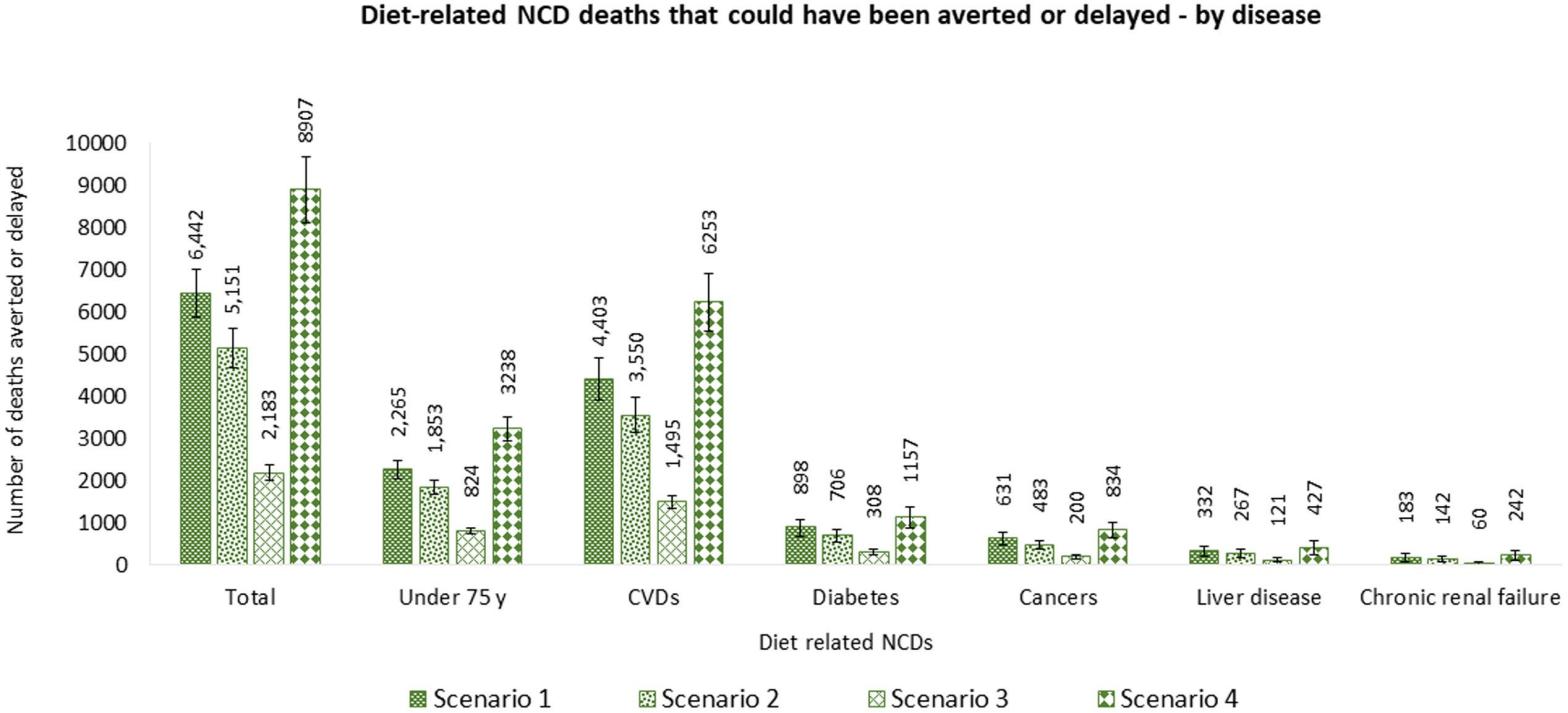
Number of diet-related NCD deaths that could be averted or delayed from implementing a ‘high in’ FOPL in Canada by disease. Potential diet-related NCD deaths that could be averted or delayed were estimated using the PRIME model (67). Inputs for the model included, (1) population demographics; (2) mortality data associated with diet-related NCDs (CVDs, diabetes, cancer, chronic renal failure, and liver disease) (2019), obtained from the publicly available Statistics Canada CANSIM tables (stratified by sex and 5-year age band) (91–96); and (3) baseline and counterfactual dietary intakes estimations using *CCHS-Nutrition 2015 PUMF data* (61, 63). Counterfactual scenario 1 and 2 were based on Taillie et al. (44). SCENARIO 1, overall changes: sodium (mg) –4.7%; sugars (g) –10.2%; saturated fat (g) –3.9%; and SCENARIO 2, disaggregated by foods: sodium (mg) –4.6%; sugars (g) –5.4%; saturated fat (g) –3.6%; and beverages: sodium (mg) –5.2%; sugars (g) –13.2%; saturated fat (g) –5.6%. Counterfactual scenario 3 was based on Acton et al. (40). SCENARIO 3, relative changes disaggregated by snack foods: sodium (mg) –6.3%; sugars (g) –0.1%; saturated fat (g) –6.5%; and beverages: sodium (mg) –5.5%; sugars (g) –8.7%;saturated fat (g) –19.5%. Counterfactual scenario 4 was based on Song et al. (41). SCENARIO 4, overall changes: sodium (mg) –7.8%; sugars (g) –7.3%; saturated fat (g) –16.3%.

Of the total diet-related NCD deaths that could have been prevented or delayed, 68.3% (4,403 [95% UI 3,916–4,892]) were related to CVDs, followed by diabetes 13.9% (898 [95% UI 684–1,088]), cancers 9.8% (631 [95% UI 493–766]), liver disease 5.2% (332 [95% UI 200–456]), and chronic renal failure 2.8% (183 [95% UI 88–272]). Furthermore, 35.2% (2,265 [95% UI 2,059–2,469] of potential deaths averted or delayed would be in individuals under 75 years old. More lives saved were predicted among men (1,532 [95% UI 1,396–1,668]) than women (733 [95% UI 649–816]) aged under 75 years (Figure 4; Supplementary Table S4.1).

#### 3.3.2. S2: Based on early evaluations of the Chilean Food Labeling and Marketing Law – changes disaggregated by food and beverages

Modeling S2 resulted in absolute mean dietary reductions of 126 mg/day sodium, 6.6 g/day sugars, 0.9 g/day saturated fats, and 35 kcal/day calories (considering only calorie contribution from changes observed in sugar and saturated fat) for adults overall (Figure 2; Supplementary Table S3). Significant differences were observed for mean sugar intakes between baseline and counterfactual S2. Stratified results by DRI age-sex group were also estimated and are presented in Supplementary Table S3.

These estimated dietary intakes changes were estimated to prevent or postpone 5,151 (95% UI 4,672–5,610) deaths from diet-related NCDs. Approximately 55% of averted or delayed deaths were in men (2,836 [95% UI 2,583–3,100]) and 45% in women (2,308 [95% UI 2,049–2,568]). Overall, more lives would have been saved in men than women (Figure 3 and Supplementary Table S4.2).

Of the total diet-related NCD deaths that could have been prevented or delayed, 68.9% (3,550 [95% UI 3,138–3,982]) were related to CVDs, followed by diabetes 13.7% (706 [95% UI 537–854]), cancers 9.4% (483 [95% UI 377–586]), liver disease 5.2% (267 [95% UI 164–363]), and chronic renal failure 2.8% (142 [95% UI 65–214]). Furthermore, 36.0% (1,853 [95% UI 1,677–2,020]) of potential deaths averted or delayed would be in individuals under 75 years old. More lives would have been saved in men (1,266 [95% UI 1,152–1,389]) than women (583 [95% UI 514–649]) aged under 75 years (Figure 4 and Supplementary Table S4.2).

#### 3.3.3. S3: Based on a Canadian randomized experimental marketplace study- changes disaggregated by snack food and beverages

Modeling S3 resulted in absolute mean dietary reductions of 31 mg/day sodium, 2.3 g/day sugars, 0.8 g/day saturated fats, and 16 kcal/day calories (considering only calorie contribution from changes observed in sugar and saturated fat) for adults overall (Figure 2 and Supplementary Table S3). Stratified results by DRI age-sex group were also estimated and are presented in Supplementary Table S3.

These estimated dietary intakes changes were estimated to prevent or postpone 2,183 (95% UI 2,008–2,361) deaths from diet-related NCDs. Approximately 57% of averted or delayed deaths were in men (1,245 [95% UI 1,148–1,340]) and 43% in women (939 [95% UI 843–1,035]) Overall, more lives would have been saved in men than women (Figure 3 and Supplementary Table S4.3).

Of the total diet-related NCD deaths that could have been prevented or delayed, 68.5% (1,495 [95% UI 1,346–1,640]) were related to CVDs, followed by diabetes 14.1% (308 [95% UI 237–372]), cancers 9.2% (200 [95% UI 156–246]), liver disease 5.5% (121 [95% UI 75–166]), and chronic renal failure 2.7% (60 [95% UI 29–90]). Furthermore, 37.7% (824 [95% UI 757–890] of potential deaths averted or delayed would be in individuals under 75 years old. More lives would have been saved in men (574 [95% UI 529–618]) than women (251 [95% UI 224–277]) aged under 75 y (Figure 4 and Supplementary Table S4.3).

#### 3.3.4. S4: Based on a meta-analysis on the impact of nutrient warning labeling – overall changes

Modeling S4 resulted in absolute mean dietary reductions of 212 mg/day sodium, 6.3 g/day sugars, 3.72 g/day saturated fats, and 59 kcal/day calories (considering only calorie contribution from changes observed in sugar and saturated fat) for adults overall (Figure 2 and Supplementary Table S3). Significant differences were observed for mean sodium, sugar, and saturated fat intakes between baseline and counterfactual S4. Stratified results by DRI age-sex group were also estimated and are presented in Supplementary Table S3.

These estimated dietary intakes changes were estimated to prevent or postpone 8,907 (95% UI 8,095–9,667) deaths from diet-related NCDs. Approximately 56% of averted or delayed deaths were in men (4,949 [95% UI 4,521-5,363]) and 44% in women (3,947 [95% UI 3,513-4,366]). Overall, more lives would have been saved in men than women (Figure 3 and Supplementary Table S4.4).

Of the total diet-related NCD deaths that could have been prevented or delayed, 70.2% (6,253 [95% UI 5,555–6,912]) were related to CVDs, followed by diabetes 13.0% (1,157 [95% UI 877–1,392]), cancers 9.4% (834 [95% UI 644–1,017]), liver disease 4.8% (427 [95% UI 253–585]), and chronic renal failure 2.7% (242 [95% UI 110–358]). Furthermore, 36.4% (3,238 [95% UI 2,952–3,518] of potential deaths averted or delayed would be in individuals under 75 years old. More lives would have been saved in men (2,237 [95% UI 2,039–2,427]) than women (1,004 [95% UI 891–1,113]) aged under 75 years (Figure 4 and Supplementary Table S4.4).

#### 3.3.5. Potential dietary and health gains by sex

Furthermore, when looking by DRI age-sex group, significant sodium reductions were observed for males ages 31–50 years (S4) and females ages 51–70 years (S4). For total sugars, significant reductions were observed in males ages 31–50 years (S1) and 51–70 years (S1), and in females ages 51–70 years (S1, S2, and S4). For saturated fat, significant changes were observed in males ages 19–30 years (S4) and 31–50 years (S4), and in females ages 31–50 years (S4), 51–70 years (S4), and > 70 years (S4).

In 2019, there were a total of 92,845 diet-related NCD deaths in Canada (men 46,568; women 46,277) (92–96); therefore 6.9% (7.5% in men, 6.4% in women), 5.5% (6.1% in men, 5.0% in women), 2.4% (2.7% in men, 2.0% in women), and 9.6% (10.6% in men, 8.5% in women) fewer diet-related NCD deaths could have been prevented or delayed under S1, S2, S3, and S4, respectively (Supplementary Table S4). Of the total number of deaths that could be prevented or delayed in all scenarios, between 87 and 92% would be attributable to changes in energy intakes (changes in obesity status), between 5 and 9% would be attributable to changes in sodium intakes, and between 1 and 6% would be attributable to changes in saturated fat intakes (Table 3).

**Table 3:** Estimated number of diet-related NCD deaths that could be averted or delayed by risk factor.

By risk factor	Scenario 1		Scenario 2		Scenario 3		Scenario 4	
	*n* (95% UI)	%	*n* (95% UI)	%	*n* (95% UI)	%	*n* (95% UI)	%
Saturated fat	59 (49, 70)	1	76 (62, 90)	1	95 (78, 113)	4	495 (411, 587)	6
Sodium	495 (211, 795)	8	485 (210, 792)	9	113 (47, 184)	5	819 (348, 1,304)	9
Calories	5,930 (5,415, 6,441)	92	4,613 (4,233, 5,006)	90	1,980 (1,815, 2,139)	91	7,710 (7,043, 8,369)	87
Total deaths *	6,442 (5,870, 7,020)	100	5,151 (4,672, 5,610)	100	2,183 (2,008, 2,361)	100	8,907 (8,095, 9,667)	100

Numbers of diet-related NCD deaths that could be averted or delayed were estimated using the PRIME model, 95% UI are based on 10,000 iterations of Monte Carlo analysis built in PRIME (67). *Total deaths averted or delayed represent less than the sum of the individual risk factors given that double counting has been accounted for in PRIME during the modeling process. Counterfactual scenario 1 and 2 were based on Taillie et al. (44). SCENARIO 1, overall changes: sodium (mg) –4.7%; sugars (g) –10.2%; saturated fat (g) –3.9%; and SCENARIO 2, disaggregated by foods: sodium (mg) –4.6%; sugars (g) –5.4%; saturated fat (g) –3.6%; and beverages: sodium (mg) –5.2%; sugars (g) –13.2%; saturated fat (g) –5.6%. Counterfactual scenario 3 was based on Acton et al. (40). SCENARIO 3, relative changes disaggregated by snack foods: sodium (mg) –6.3%; sugars (g) –0.1%; saturated fat (g) –6.5%; and beverages: sodium (mg) –5.5%; sugars (g) –8.7%; saturated fat (g) –19.5%. Counterfactual scenario 4 was based on Song, et al. (41). SCENARIO 4, overall changes: sodium (mg) –7.8%; sugars (g) –7.3%; saturated fat (g) –16.3%.

### 3.4. Sensitivity analysis

Sensitivity analysis, using changes observed in calories overall and not only calorie contribution from changes observed in sugar and saturated fat, as the main analysis, resulted in absolute mean calorie reductions of 63, 44, 31, and 232 kcal/day in S1, S2, S3, and S4, respectively. Health gains would have been greater under all scenarios tested. For instance, the number of deaths that could have been prevented or delayed increased by 35% under S1, 22% under S2, 94% under S3, and by 252% under S4 (Supplementary Table S4.1.1, S4.2.1, S4.3.1, and S4.4.1).

Modeling criteria that WHO (20) used to estimate FOPL cost-effectiveness resulted in absolute mean dietary reductions of 174 mg/day of sodium, 2.9 g/day of saturated fats, and 95 kcal/day of calories. Calorie reductions in this scenario were modeled directly from WHO criteria, as reduction for sugars was not included (Supplementary Table S5). Significant differences were observed for mean sodium, saturated fat, and calorie intakes between baseline and the WHO counterfactual scenario. These estimated dietary intakes changes were estimated to prevent or postpone 12,416 (95% UI 11,351–13,469) deaths from diet-related NCDs. Approximately 56% of averted or delayed deaths were in men (6,960 [95% UI 6,383–7,512]) and 44% in women (5,486 [95% UI 4,839–6,095]). Overall, more lives would have been saved in men than women (Supplementary Table S5.1).

## 4. Discussion

This is the first study to model the potential dietary and health impacts of implementing a ‘high in’ FOPL symbol in Canada. Data from *CCHS-Nutrition 2015 PUMF* was used to estimate the baseline sodium, sugars, saturated fat, and calorie intakes of Canadian adults, to then model the potential reductions in these nutrients and calorie intakes, and resulting health outcomes, based on reductions observed in recent experimental and observational studies examining ‘high in’ FOPL systems.

Our results show that current calorie and nutrient intakes estimations (baseline scenario) are consistent with previous estimations using *CCHS-Nutrition 2015* (2, 4, 107), showing, for example, that Canadian adults’ average sodium intake (2,729 mg/day) is well above the recommended level of 2,300 mg/day (2). Furthermore, after comparing intakes between the baseline scenario and modeled FOPL counterfactual scenarios, our estimates suggest that ‘high in’ FOPL could significantly reduce Canadian adults’ intakes of sodium (S1and S4), total sugars (S1, S2, and S4), and saturated fat (S4). These changes could be attributable to both healthier food choices (i.e., food substitution with a healthier, less healthy, or similar alternatives; not changing their purchase behavior in response of the ‘high in’ FOPL for some products, abandoning consumption of the product, or increasing consumption of fresh produce or minimally processed foods) in response to the FOPL (S1, S2, S3, and S4), as well as industry-driven initial food reformulation in the presence of a ‘high in’ FOPL policy (S1 and S2). These results demonstrate the potential impact of FOPL on reducing intakes of nutrients of public health concern, of which consumption remains at high levels in Canada (2–7).

The ‘high in’ FOPL symbol has previously been proven to be effective at helping Canadians with different levels of health literacy to identify foods high in nutrients to limit and make healthier food choices; and has shown that with increased exposure and awareness to the ‘high in’ symbol consumers became more efficient at choosing healthier food options (59). Additionally, evidence has shown that Canadian consumers specifically preferred FOPL that highlights individual nutrients to limit, in line with the current policy (108, 109). This evidence supports the hypothesis that consumers will in fact adopt and use the ‘high in’ FOPL symbol, indicating that the potential dietary outcomes estimated in this study could be viable. Moreover, while it remains to be examined, previous evidence has shown that the reduction of nutrients to limit due to FOPL could potentially lead to improving dietary intakes of nutrients to encourage, such as fiber (110), furthering the potential health impacts.

In Canada, the current burden of diet-related NCDs is of concern. For instance, CVDs are the second leading cause of death (1), 23% of adults have hypertension (111), 63% of adults are affected by either obesity or overweight (112), and 30% of Canadians are currently living with diabetes or pre-diabetes (113). It has been well established that high intake of sodium increase risk for hypertension, CVDs, stroke, and renal disease (114–117). Also, caloric contribution from excess sugar intakes has been associated with adverse health effects such as CVDs and diabetes (118–121). The PRIME model estimated that most of the health gains from estimated dietary intake changes were from CVDs (~70%), followed by diabetes, cancers, liver disease, and chronic renal failure, indicating that FOPL could in fact have a positive impact on the most problematic diet-related NCDs in Canada. The greatest differences by sex were observed for ischemic heart disease, in all scenarios modeled, where number of deaths that could be prevented or delayed for men almost doubles estimated numbers for women. This could be explained, in part, by differences in dietary intakes (2, 4, 107) and disease-specific mortality burden between males and females (91–96). Additionally, in most policy scenarios modeled (S2–S4), more lives would have been saved in men than women, overall, and in the population aged less than 75 years. Almost 36% of diet-related NCD deaths that could be prevented or delayed would occur in people under 75 (premature deaths). To put this in context, the number of diet-related NCD deaths averted or delayed estimated in this study would represent 6.9% (S1), 5.5% (S2), 2.4% (S3), and 9.6% (S4) of diet-related NCD deaths reported in 2019 in Canada (91–96).

Most of the estimated health gains of implementing a ‘high in’ FOPL in Canada (1,980 – 7,710; ~90%) would be attributable to estimated changes in energy intake (between 16 kcal/day and 59 kcal/day). This results from simulating a range of policy scenarios that goes from assessing impact of ‘high in’ FOPL only in snack foods and beverages (S3) to all food and beverages (S1, S2, and S4) consumed by Canadian adults. Our results provide similar and, in some scenarios, more conservative estimates when compared with previous studies. For instance, substituting foods that would carry a red traffic light FOPL (UK system) with foods without a red traffic light FOPL, an idealistic response to FOPL, could avert or delay 11,715 (10,500–12,865) deaths from diet-related NCDs in Canada (90). Many of the estimated deaths prevented or delayed were also attributable to reductions in mean calorie intake (90%), which was reduced on average by 122 kcal/day for men and 90 kcal/day for women (90). Also, introducing a ‘high in’ FOPL in Mexico was estimated to yield a reduction of 36.8 kcal/day, and this resulted in a reduction of 4.98 percentage points in obesity prevalence among Mexican adults (122). The study considered reductions in calorie intakes from snack foods and beverages using Acton et al. (40) estimations, similar to our counterfactual S3. However, to be conservative, we estimated changes in calorie intakes by reducing only calorie contribution from changes observed in sugar and saturated fat from Acton et al. (40). This resulted in a modest reduction of 16 kcal/day and an estimated reduction of 2.4% of diet-related NCD deaths that could be averted or delayed. Sensitivity analyses, using changes observed in calories overall for S3, yielded reductions of 31 kcal/day [similar to the study in Mexico (122)] and an estimated reduction of 4.6% of diet-related NCD deaths that could be averted or delayed among Canadian adults, using as a reference Canadian diet-related NCD mortality for 2019. Even though some of the modeled nutrient and calorie reductions may appear modest, they could translate to meaningful improvements in health outcomes at a population level.

Overall, our results are consistent with previous estimations in terms of potential health gains attributable to improving diet quality overall (90, 122, 123), and contribute to the body of evidence supporting policies to improve the Canadian food environment, specifically implementation of a ‘high in’ FOPL symbol. However, the addition of other proven cost-effective policies should be considered to improve diets in Canada, such as moving from voluntary to mandatory sodium reduction targets and implementing SSB taxes, as evidence shows these initiatives could lead to further changes in purchasing behavior, health gains, and health care cost savings (124–126). For example, in Canada, introducing a 20% tax on SSBs would potentially reduce Canadian adults’ energy intake by 21 kcal/day for men and 13 kcal/day for women, which could postpone 7,874 (6,630–9,118) deaths, mainly from ischemic heart disease or cancer (123); and fully meeting voluntary sodium reduction targets for processed foods would potentially reduce mean sodium intakes by 459 mg/day, which could avert or delay 2,176 (95% UI 869–3,687) deaths from CVDs (3).

As mentioned earlier, the purpose of a FOPL system is to assess the nutritional quality of food products and display it in a simple, easy to interpret visual form to help consumers make informed food choices and motivate the food industry to reformulate food products (12). Focusing on reducing a few nutrients of public health concern could indeed motivate industry driven reformulation to produce products that contain less of these nutrients, as evidence suggests (45–48). However, this does not necessarily mean healthier food products will be introduced. For instance, previous studies monitoring the Canadian food supply have shown that a reduction in sugar levels does not seem to necessarily translate into lower energy content due to an increase in other nutrients (e.g., fats and starches) for some food categories (127). This could have unintended consequences such as not leading to an equivalent reduction in food caloric content, or even leading to an increase in caloric content. Moreover, evidence from Chile shows that non-nutritive sweetener intakes among preschoolers significantly increased after the implementation of the first phase of the Chilean Food Labeling and Marketing Law (128). Therefore, it is imperative to accompany implementation of policies – such as ‘high in’ FOPL – with robust and independent evaluation and monitoring systems to monitor effectiveness and compliance with the policy, but also to detect and correct any unintended consequences. This is particularly important given that over 60% of packaged foods in the Canadian food supply would carry a ‘high in’ symbol (129). Additional measures could also be taken to mitigate possible unintended consequences following examples from other countries, for instance, flagging products containing non-nutritive sweeteners as Mexico (52) and Argentina (53) have done in their FOPL regulations.

Several strengths and limitations should be considered in the interpretation of our results. This was the first study to use WHO endorsed methods to estimate the impact of a ‘high in’ FOPL policy using recent nationally representative data and several counterfactual scenarios. A key strength of our study includes using data from the nationally representative *CCHS-Nutrition 2015* survey to estimate our baseline and counterfactual scenarios. Given the nature of the survey there could be inherent issues with misreporting; however, the survey used the Automated Multiple Pass Method to minimize misreporting bias (61). Additionally, we used both 24 h recall days and the NCI method to assess usual calorie and nutrient intakes for Canadian adults, overall and stratified by DRI age-sex group, adjusting for several relevant covariates. This allowed for the input of disaggregated data, by DRI age-sex group, in the PRIME model.

Our study was also strengthened by the use of PRIME, a macrosimulation model that has been used extensively (69–88), and uses relative risks from robust meta-analyses (67). However, as a cross-sectional NCD scenario model, PRIME does not incorporate the effect of time lag between the exposure and disease outcome. Additionally, the model does not study morbidity that could be prevented from changes in the NCD behavioral risk factors of interest. Strengths and limitations of the PRIME model have been discussed in more detail elsewhere (67).

Counterfactual scenarios modeled in this study were based on the most recent available evidence on changes in food and beverage purchases in the presence of a ‘high in’ FOPL. There is scarce evidence of the impact of FOPL policies on dietary intakes; thus, it was assumed that observed changes in calorie and critical nutrients of food and beverage purchases in the presence of a ‘high in’ FOPL would transpose to dietary intakes – as research comparing food purchase and 24 h recall data suggests that documented food purchases can act as reasonably accurate estimates of overall diet quality (130). Additionally, we provided conservative estimates given that changes in calorie intakes were calculated from reducing only calorie contribution from changes observed in sugar and saturated fat, instead of directly applying changes observed in calories overall.

We modeled different policy scenarios, for instance, using the evidence from Chile that, to best of our knowledge, is the only country to date that has evaluated changes in food and beverages purchases before and after FOPL policy implementation. Changes observed in Chile in response to FOPL may not necessarily translate into similar outcomes in Canada given differences in dietary patterns, cultural food norms, and food environments between the two countries; however, the Chilean and Canadian populations face similar diet-related risk factors. For instance, both countries have one of the highest retail sales *per capita* of ultra-processed products (131), high sodium intakes (2, 132), and face high prevalence of obesity (133, 134). Also, it is worth noting that the comprehensive Chilean Food Labeling and Marketing Law, not only includes a mandatory ‘high in’ warning label, but also restricts sales and promotion in schools, and marketing to children for foods that exceed established thresholds for calories and targeted nutrients (133) – measures that have not yet been implemented in Canada. Therefore, it is more likely that first evaluations of the Chilean Law are also a result of these other components of the regulation. However, greater changes are expected after the implementation of phases 2 and 3 of the Law with more stringent thresholds, which makes this a conservative counterfactual scenario to model. Furthermore, early phase 1 Chilean thresholds, applied in scenarios 1 and 2, were generally less stringent than those in the Canadian regulations (12, 42). However, policy scenarios that incorporate trends in consumer purchase behavior after implementation of FOPL should be explored further when more evidence on FOPL impacts overtime is available; specifically, it remains to be seen if the effects on consumer food choices plateaus over time, and if so, at what level.

Lastly, our most conservative scenario, where reductions were only applied to snack foods and beverages, was based on a randomized experimental marketplace study (40) conducted among Canadian adults, which reflects the preferences of our Canadian population. Additionally, we also used evidence from a meta-analysis of randomized controlled trials and quasi experimental studies examining overall changes in consumer food and beverage purchases that lead to a decrease in the content of critical nutrients and calories (41). Nevertheless, future natural experiments evaluating the impact of the Canadian ‘high in’ FOPL symbol on food choices, diet quality, and health outcomes will be needed to corroborate estimations from this study.

## 5. Conclusion

Results from this study suggest that implementation of a ‘high in’ FOPL symbol could significantly reduce sodium, total sugar, and saturated fat intakes among Canadian adults. These dietary changes could prevent or postpone up to 8,907 diet-related NCD deaths in Canada, primarily from CVDs. Future studies should continue this work to estimate the cost-effectiveness of this policy in the Canadian context and investigate potential differential impacts across diversity subgroups. Overall, these results provide critical evidence to inform and support policy implementation of the recently published ‘high in’ FOPL regulations in Canada.

## Data availability statement

Publicly available datasets were analyzed in this study. Canadian Community Health Survey-Nutrition 2015 Public Use Microdata File (PUMF) data is publicly and freely available without restriction at Statistics Canada, https://www150.statcan.gc.ca/n1/en/catalogue/82M0024X. Analytic code (SAS) can be made available to researchers upon request to the author. Canadian population demographics and data on mortality associated with diet-related NCDs (CVDs, diabetes, cancer, chronic renal failure, and liver disease) — stratified by sex and 5-year age band — were obtained from the publicly available Statistics Canada CANSIM tables (2019). https://www150.statcan.gc.ca/t1/tbl1/en/tv.action?pid=1710000501, https://www150.statcan.gc.ca/t1/tbl1/en/tv.action?pid=1310014201, https://www150.statcan.gc.ca/t1/tbl1/en/tv.action?pid=1310014401, https://www150.statcan.gc.ca/t1/tbl1/en/tv.action?pid=1310014701, https://www150.statcan.gc.ca/t1/tbl1/en/tv.action?pid=1310015101, https://www150.statcan.gc.ca/t1/tbl1/en/tv.action?pid=1310014801.

## Author contributions

NF, NK, RA, and ML’A conceptualized the study design. NF, AN, and MA conducted the study. NF, MA, and JL analyzed and interpreted the data. All authors critically reviewed and approved the final manuscript.

## Funding

This research was funded by Canadian Institutes of Health Research (CIHR) operating grants (PJT-165858 and SA2-152805; https://cihr-irsc.gc.ca/e/193.html). The funders had no role in study design, data collection and analysis, decision to publish, or preparation of the manuscript.

## Conflict of interest

The authors declare that the research was conducted in the absence of any commercial or financial relationships that could be construed as a potential conflict of interest.

## Publisher’s note

All claims expressed in this article are solely those of the authors and do not necessarily represent those of their affiliated organizations, or those of the publisher, the editors and the reviewers. Any product that may be evaluated in this article, or claim that may be made by its manufacturer, is not guaranteed or endorsed by the publisher.
