# Supplementary material for: Estimating the dietary and health impact of implementing front-of-pack nutrition labeling in Canada: A macrosimulation modeling study
Source: Front Nutr. 2023 Mar 17;10:1098231. doi: 10.3389/fnut.2023.1098231 (PMC10065472; doi:10.3389/fnut.2023.1098231)
Supplement: Supplementary file 1 [file Data_Sheet_1.pdf]

Supplemental material

Estimating the dietary and health impact of implementing front-of-pack nutrition labeling in Canada: A macrosimulation modeling study

Nadia Flexner[1], Alena P. Ng[1], Mavra Ahmed[1,2], Neha Khandpur[3,4,5], Rachel B. Acton[6], Jennifer J. Lee[1], Mary R. L'Abbe[1*]

[1]Department of Nutritional Sciences, Temerty Faculty of Medicine, University of Toronto, Toronto, ON, Canada.
[2]Joannah and Brian Lawson Centre for Child Nutrition, Temerty Faculty of Medicine, University of Toronto, Toronto, ON, Canada.
[3] Department of Human Nutrition and Health, Wageningen University, Wageningen, Netherlands.
[4]Center for Epidemiological Research in Nutrition and Health, School of Public Health, University of São Paulo, São Paulo, Brazil.
[5]Department of Nutrition, Harvard T. H. Chan School of Public Health, Harvard University, Boston, MA, United States.
[6] School of Public Health and Health Systems, University of Waterloo, Waterloo, ON, Canada.

[*]Corresponding author: mary.labbe@utoronto.ca

Table S1. Snack food and beverages grouped by Health Canada's TRA categories, used in Scenario 3.

Name	Examples	Matching TRA Minor Categories*
**Snack foods (Scenario 3)**		
Cakes, cookies, and other baked goods	Brownies, cakes, coffee cakes, donuts, Danishes, sweet rolls	A.4, A.5, A.6, A.7, A.8, A.9, A.10, A.14, A.21, A.22, A.23
Crackers	Accompaniment crackers, hard bread sticks, melba toast, snack crackers, dry breads, rusks	A.11, A.12, A.13
Granola and energy bars	Grain-based bars, energy bars, protein bars	A.18, A.19, A.20
Cheese	Cream cheese, cheese spread, flavoured cheese, shredded cheese	D.1
Yogurt	Yogurt in tubs, drinkable yogurt, kefir	D.12, D.15
Frozen desserts	Ice cream, ice milk, frozen yogurt, sherbet, ice cream cone	E.1, E.2, E.3, E.4, A.15
Custard and pudding	Custard, gelatin, pudding	E.5
Chocolate and candies	Confectionaries, chocolate, candies, gum, mints, gummies, marshmallow, halva	M.7, U.1, U.2, U.3, U.4, U.5, U.6, U.10, U.11, O.2
Canned, fresh, and frozen fruits	All fruits (including avocados), lemon/lime juices used as ingredients	J.1, J.2, J.3, J.4, J.5, J.12
Prepared fruits	Applesauce, dried fruit, pickled fruit, fruit for garnish, fruit relishes	J.6, J.7, J.8, J.9, J.10, M.12
Canned, fresh, and frozen vegetables	Fresh, frozen, canned non-starchy vegetables with and without sauce	V.1, V.2, V.3, V.4, V.5, V.6
Potato chips, popcorn, pretzels, and other chips	Potato chips, corn chips, rice chips, pretzels, pulse-based chips, fruit chips, pita chips	S.1
Nuts and seeds	Coated and uncoated, mixes with chocolate and dried fruit	S.2, O.1
Meat snacks	Jerky	S.3
**Beverages (Scenario 3)**		
Carbonated and non-carbonated beverages	Sugar-sweetened beverages, artificially sweetened beverages, energy drinks, water, vitamin water, coconut water, cocoa and chocolate beverages, non-alcoholic beverages	B.1, B.5
Coffee & Tea	Sweetened and unsweetened coffee & tea	B.3, B.4
Milk and substitutes	Milk (all fat levels), plant-based dairy substitutes (inc. Almonds, cashew, soy, coconut)	D.11
Fruit juices and drinks	Fruit juices, nectars, fruit drinks	J.11

*Snack foods and beverages were grouped in fourteen and four food categories, respectively, using Health Canada's Table of References Amounts (1).

Table S2. Baseline scenario: Canadian adults' (≥19 y) calorie and targeted nutrient intakes by DRI age/sex group, overall and by food and beverage contribution (n=11,992)

CURRENT CALORIE & TARGETED NUTRIENT INTAKES (≥19 y)																
	Energy (kcal/d)	*SE*	*Food*	*Beverage*	***Sodium (mg/d)***	*SE*	*Food*	*Beverage*	***Sugars (g/d)***	*SE*	*Food*	*Beverage*	***Sat fats (g/d)***	*SE*	*Food*	*Beverage*
Total 19+	***1889***	*20*	*1634*	*255*	***2729***	*33*	*2572*	*157*	***86.43***	*0.95*	*59.94*	*27.35*	***22.84***	*0.56*	*21.07*	*1.98*
Males																
19-30 y	**2373**	51	2019	343	**3435**	85	3234	195	**101.36**	3.49	62.98	37.10	**28.87**	0.82	26.65	2.10
31-50 y	**2236**	36	1911	317	**3247**	66	3061	181	**96.58**	2.11	63.07	32.86	**27.01**	0.71	24.95	2.07
51-70 y	**2039**	54	1755	284	**2974**	86	2807	164	**90.31**	2.38	62.79	28.45	**24.39**	1.13	22.55	2.06
> 70 y	**1905**	88	1649	259	**2775**	126	2623	150	**86.01**	4.41	63.32	24.96	**22.56**	1.60	20.87	2.03
Females																
19-30 y	**1710**	78	1488	223	**2448**	122	2301	151	**81.02**	5.02	53.95	26.59	**20.83**	1.00	19.12	1.82
31-50 y	**1665**	32	1455	214	**2387**	52	2247	145	**80.14**	2.21	56.27	24.69	**20.28**	0.38	18.65	1.89
51-70 y	**1585**	25	1394	200	**2272**	31	2141	135	**77.88**	1.20	58.17	22.20	**19.30**	0.61	17.77	1.93
> 70 y	**1464**	36	1297	182	**2107**	45	1987	124	**73.86**	2.32	58.81	19.29	**17.74**	0.80	16.35	1.95

Baseline and counterfactual calorie and nutrient intakes were estimated using *CCHS-Nutrition 2015 PUMF data* (2, 3). Usual intakes were estimated using the National Cancer Institute (NCI) method, and analyses were adjusted for age, sex, dietary misreporting status, weekend/weekday, and sequence of dietary recall. Abbreviations: d=day; g=grams; mg=milligrams; kcal=kilocalories; SE=standard error.

Table S3. Counterfactual scenario: Canadian adults' (≥19 y) calorie and targeted nutrient intakes, by FOPL counterfactual scenario tested (n=11,992)

Table S3.1. Potential changes in usual energy intake of implementing a 'high in' FOPL symbol in Canada (≥19 y), overall and by DRI age/sex group. Counterfactual energy intake considering reductions of calorie contribution from changes observed in sugar and saturated fat.

ENERGY (kcal/d)															
	n	***Baseline mean***	*SE*	***S1 mean***	*SE*	*S1 Δ*	***S2 mean***	*SE*	*S2 Δ*	***S3 mean***	*SE*	*S3 Δ*	***S4 mean***	*SE*	*S4 Δ*
Total 19+	***11991***	***1889***	*20*	***1845***	*20*	***43***	***1854***	*20*	***35***	***1873***	*20*	***16***	***1830***	*19*	***59***
Males															
19-30 y	765	**2373**	51	**2321**	50	**52**	**2329**	50	**44**	**2351**	50	**22**	**2300**	49	**73**
31-50 y	1839	**2236**	36	**2186**	36	**50**	**2195**	36	**41**	**2216**	36	**20**	**2168**	35	**68**
51-70 y	1964	**2039**	54	**1994**	53	**46**	**2003**	53	**36**	**2023**	54	**16**	**1977**	52	**62**
> 70 y	1105	**1905**	88	**1862**	86	**43**	**1872**	86	**33**	**1891**	87	**14**	**1847**	85	**58**
Females															
19-30 y	815	**1710**	78	**1670**	76	**40**	**1678**	76	**33**	**1694**	77	**16**	**1656**	75	**54**
31-50 y	2056	**1665**	32	**1625**	31	**39**	**1633**	31	**31**	**1650**	32	**15**	**1612**	31	**53**
51-70 y	2107	**1585**	25	**1547**	24	**38**	**1556**	24	**29**	**1573**	24	**13**	**1535**	24	**51**
> 70 y	1340	**1464**	36	**1429**	34	**35**	**1438**	35	**26**	**1454**	35	**11**	**1418**	34	**47**

Table S3.1.1. Potential changes in usual energy intake of implementing a 'high in' FOPL symbol in Canada (≥19 y), overall and by DRI age/sex group. Sensitivity analysis, considering percentage reductions in calories from evidence that was used to construct FOPL counterfactual scenarios.

ENERGY (kcal/d)

	n	***Baseline mean***	*SE*	***S1 mean***	*SE*	*S1 Δ*	***S2 mean***	*SE*	*S2 Δ*	***S3 mean***	*SE*	*S3 Δ*	***S4 mean***	*SE*	*S4 Δ*
Total 19+	***11991***	***1889***	*20*	***1826***	*20*	***63***	***1844***	*20*	***44***	***1858***	*20*	***31***	***1657****	*19*	***232***
Males															
19-30 y	765	**2373**	51	**2294**	49	**79**	**2315**	50	**57**	**2334**	50	**39**	**2083***	45	**290**
31-50 y	1839	**2236**	36	**2162**	35	**74**	**2183**	35	**53**	**2200**	36	**36**	**1963***	32	**273**
51-70 y	1964	**2039**	54	**1972**	53	**68**	**1992**	53	**47**	**2008**	53	**31**	**1791***	49	**249**
> 70 y	1105	**1905**	88	**1842**	86	**63**	**1862**	86	**43**	**1877**	86	**28**	**1673**	78	**232**
Females															
19-30 y	815	**1710**	78	**1652**	75	**58**	**1669**	76	**42**	**1680**	77	**30**	**1497**	68	**213**
31-50 y	2056	**1665**	32	**1608**	31	**56**	**1625**	31	**40**	**1636**	31	**28**	**1458***	27	**207**
51-70 y	2107	**1585**	25	**1532**	24	**53**	**1549**	24	**37**	**1559**	24	**26**	**1389***	22	**196**
> 70 y	1340	**1464**	36	**1415**	35	**49**	**1432**	35	**33**	**1441**	35	**23**	**1284***	32	**181**

Baseline and counterfactual calorie and nutrient intakes were estimated using *CCHS-Nutrition 2015 PUMF data* (2, 3). Usual intakes were estimated using the National Cancer Institute (NCI) method, and analyses were adjusted for age, sex, dietary misreporting status, weekend/weekday, and sequence of dietary recall. Abbreviations: d=day; g=grams; mg=milligrams; kcal=kilocalories; SE=standard error; FOPL= front-of-pack labeling; S1=scenario 1; S2=scenario 2; S3=scenario 3; S4=scenario 4. Baseline and counterfactual scenarios are described in methods. Counterfactual scenario 1 and 2 were based on Taillie, et. al. (4). **SCENARIO 1**, overall changes: sodium (mg) -4.7%; sugars (g) -10.2%; saturated fat (g) -3.9%; and **SCENARIO 2**, disaggregated by foods: sodium (mg) -4.6%; sugars (g) -5.4%; saturated fat (g) -3.6%; and beverages: sodium (mg) -5.2%; sugars (g) -13.2%; saturated fat (g) -5.6%. Counterfactual scenario 3 was based on Acton, et. al. (5). **SCENARIO 3**, relative changes disaggregated by snack foods: sodium (mg) -6.3%; sugars (g) -0.1%; saturated fat (g) -6.5%; and beverages: sodium (mg) -5.5%; sugars (g) -8.7%; saturated fat (g) -19.5%. Counterfactual scenario 4 was based on Song, et. al. (6). **SCENARIO 4**, overall changes: sodium (mg) -7.8%; sugars (g) -7.3%; saturated fat (g) -16.3%**.** * Indicates a statistically significant difference between baseline mean intakes and counterfactual mean intakes.

Table S3.2. Potential changes in usual sodium intake of implementing a 'high in' FOPL symbol in Canada (≥19 y), overall and by DRI age/sex group.

SODIUM (mg/d)															
	n	***Baseline mean***	*SE*	***S1 mean***	*SE*	*S1 Δ*	***S2 mean***	*SE*	*S2 Δ*	***S3 mean***	*SE*	*S3 Δ*	***S4 mean***	*SE*	*S4 Δ*
Total 19+	***11992***	***2729***	*33*	***2602****	*32*	***128***	***2603***	*32*	***126***	***2699***	*33*	***31***	***2517****	*31*	***212***
Males															
19-30 y	765	**3435**	85	**3274**	81	**161**	**3276**	81	**158**	**3397**	84	**38**	**3168**	79	**267**
31-50 y	1839	**3247**	66	**3095**	63	**152**	**3097**	63	**150**	**3212**	65	**35**	**2995***	61	**252**
51-70 y	1965	**2974**	86	**2834**	82	**139**	**2836**	82	**137**	**2942**	85	**31**	**2743**	80	**231**
> 70 y	1105	**2775**	126	**2645**	120	**130**	**2647**	120	**128**	**2747**	123	**28**	**2560**	116	**216**
Females															
19-30 y	815	**2448**	122	**2334**	116	**115**	**2335**	116	**113**	**2418**	121	**30**	**2258**	112	**190**
31-50 y	2056	**2387**	52	**2275**	49	**112**	**2277**	49	**110**	**2358**	51	**29**	**2202**	47	**186**
51-70 y	2107	**2272**	31	**2165**	29	**106**	**2167**	29	**105**	**2245**	30	**27**	**2095***	28	**177**
> 70 y	1340	**2107**	45	**2008**	43	**99**	**2010**	43	**97**	**2083**	44	**25**	**1943**	42	**164**

Baseline and counterfactual calorie and nutrient intakes were estimated using *CCHS-Nutrition 2015 PUMF data* (2, 3). Usual intakes were estimated using the National Cancer Institute (NCI) method, and analyses were adjusted for age, sex, dietary misreporting status, weekend/weekday, and sequence of dietary recall. Abbreviations: d=day; g=grams; mg=milligrams; kcal=kilocalories; SE=standard error; FOPL= front-of-pack labeling; S1=scenario 1; S2=scenario 2; S3=scenario 3; S4=scenario 4. Baseline and counterfactual scenarios are described in methods. Counterfactual scenario 1 and 2 were based on Taillie, et. al. (4). **SCENARIO 1**, overall changes: sodium (mg) -4.7%; sugars (g) -10.2%; saturated fat (g) -3.9%; and **SCENARIO 2**, disaggregated by foods: sodium (mg) -4.6%; sugars (g) -5.4%; saturated fat (g) -3.6%; and beverages: sodium (mg) -5.2%; sugars (g) -13.2%; saturated fat (g) -5.6%. Counterfactual scenario 3 was based on Acton, et. al. (5). **SCENARIO 3**, relative changes disaggregated by snack foods: sodium (mg) -6.3%; sugars (g) -0.1%; saturated fat (g) -6.5%; and beverages: sodium (mg) -5.5%; sugars (g) -8.7%; saturated fat (g) -19.5%. Counterfactual scenario 4 was based on Song, et. al. (6). **SCENARIO 4**, overall changes: sodium (mg) -7.8%; sugars (g) -7.3%; saturated fat (g) -16.3%**.** * Indicates a statistically significant difference between baseline mean intakes and counterfactual mean intakes.

Table S3.3. Potential changes in usual total sugar intake of implementing a 'high in' FOPL symbol in Canada (≥19 y), overall and by DRI age/sex group.

SUGARS (g/d)

	n	***Baseline mean***	*SE*	***S1 mean***	*SE*	*S1 Δ*	***S2 mean***	*SE*	*S2 Δ*	***S3 mean***	*SE*	*S3 Δ*	***S4 mean***	*SE*	*S4 Δ*
Total 19+	***11988***	***86.43***	*0.95*	***77.70****	*0.85*	***8.74***	***79.79****	*0.86*	***6.64***	***84.18***	*0.91*	***2.26***	***80.18****	*0.88*	***6.25***
Males															
19-30 y	765	**101.36**	3.49	**91.07**	3.14	**10.29**	**93.05**	3.19	**8.31**	**98.17**	3.37	**3.19**	**93.99**	3.24	**7.37**
31-50 y	1837	**96.58**	2.11	**86.80***	1.90	**9.79**	**88.89**	1.93	**7.69**	**93.79**	2.04	**2.80**	**89.58**	1.96	**7.00**
51-70 y	1964	**90.31**	2.38	**81.19***	2.15	**9.13**	**83.39**	2.20	**6.93**	**87.98**	2.33	**2.33**	**83.78**	2.22	**6.53**
> 70 y	1105	**86.01**	4.41	**77.33**	3.98	**8.67**	**79.65**	4.08	**6.35**	**84.05**	4.31	**1.96**	**79.80**	4.10	**6.21**
Females															
19-30 y	815	**81.02**	5.02	**72.81**	4.50	**8.21**	**74.64**	4.60	**6.38**	**78.73**	4.86	**2.29**	**75.15**	4.65	**5.88**
31-50 y	2056	**80.14**	2.21	**72.04**	1.97	**8.10**	**74.03**	2.02	**6.11**	**78.10**	2.14	**2.05**	**74.35**	2.04	**5.80**
51-70 y	2107	**77.88**	1.20	**70.03***	1.09	**7.85**	**72.18***	1.12	**5.70**	**76.14**	1.18	**1.73**	**72.26***	1.12	**5.61**
> 70 y	1339	**73.86**	2.32	**66.44**	2.10	**7.42**	**68.69**	2.17	**5.17**	**72.47**	2.29	**1.39**	**68.55**	2.17	**5.31**

Baseline and counterfactual calorie and nutrient intakes were estimated using *CCHS-Nutrition 2015 PUMF data* (2, 3). Usual intakes were estimated using the National Cancer Institute (NCI) method, and analyses were adjusted for age, sex, dietary misreporting status, weekend/weekday, and sequence of dietary recall. Abbreviations: d=day; g=grams; mg=milligrams; kcal=kilocalories; SE=standard error; FOPL= front-of-pack labeling; S1=scenario 1; S2=scenario 2; S3=scenario 3; S4=scenario 4. Baseline and counterfactual scenarios are described in methods. Counterfactual scenario 1 and 2 were based on Taillie, et. al. (4). **SCENARIO 1**, overall changes: sodium (mg) -4.7%; sugars (g) -10.2%; saturated fat (g) -3.9%; and **SCENARIO 2**, disaggregated by foods: sodium (mg) -4.6%; sugars (g) -5.4%; saturated fat (g) -3.6%; and beverages: sodium (mg) -5.2%; sugars (g) -13.2%; saturated fat (g) -5.6%. Counterfactual scenario 3 was based on Acton, et. al. (5). **SCENARIO 3**, relative changes disaggregated by snack foods: sodium (mg) -6.3%; sugars (g) -0.1%; saturated fat (g) -6.5%; and beverages: sodium (mg) -5.5%; sugars (g) -8.7%; saturated fat (g) -19.5%. Counterfactual scenario 4 was based on Song, et. al. (6). **SCENARIO 4**, overall changes: sodium (mg) -7.8%; sugars (g) -7.3%; saturated fat (g) -16.3%**.** * Indicates a statistically significant difference between baseline mean intakes and counterfactual mean intakes.

Table S3.4. Potential changes in usual saturated fat intake of implementing a 'high in' FOPL symbol in Canada (≥19 y), overall and by DRI age/sex group.

SATURATED FAT (g/d)																
	**n**	**Baseline mean**	*SE*	**S1 mean**	*SE*	*S1 Δ*	**S2 mean**	*SE*	*S2 Δ*	**S3 mean**	*SE*	*S3 Δ*	**S4 mean**	*SE*	*S4 Δ*
Total 19+	***11991***	***22.84***	*0.56*	***21.95***	*0.54*	***0.89***	***21.98***	*0.54*	***0.86***	***22.08***	*0.54*	***0.76***	***19.12****	*0.47*	***3.72***
Males															
19-30 y	765	**28.87**	0.82	**27.75**	0.79	**1.12**	**27.79**	0.79	**1.08**	**27.94**	0.79	**0.93**	**24.18***	0.69	**4.69**
31-50 y	1839	**27.01**	0.71	**25.96**	0.68	**1.05**	**26.00**	0.68	**1.01**	**26.14**	0.68	**0.87**	**22.61***	0.59	**4.40**
51-70 y	1964	**24.39**	1.13	**23.44**	1.09	**0.95**	**23.48**	1.09	**0.91**	**23.61**	1.09	**0.78**	**20.42**	0.95	**3.97**
> 70 y	1105	**22.56**	1.60	**21.68**	1.54	**0.88**	**21.71**	1.54	**0.85**	**21.84**	1.54	**0.72**	**18.89**	1.34	**3.67**
Females															
19-30 y	815	**20.83**	1.00	**20.02**	0.96	**0.81**	**20.04**	0.97	**0.79**	**20.09**	0.97	**0.74**	**17.44**	0.84	**3.39**
31-50 y	2056	**20.28**	0.38	**19.49**	0.37	**0.79**	**19.52**	0.37	**0.76**	**19.57**	0.37	**0.71**	**16.98***	0.32	**3.30**
51-70 y	2107	**19.30**	0.61	**18.55**	0.59	**0.75**	**18.57**	0.59	**0.73**	**18.62**	0.59	**0.68**	**16.16***	0.51	**3.14**
> 70 y	1340	**17.74**	0.80	**17.04**	0.77	**0.70**	**17.07**	0.77	**0.67**	**17.12**	0.77	**0.62**	**14.85***	0.67	**2.89**

Baseline and counterfactual calorie and nutrient intakes were estimated using *CCHS-Nutrition 2015 PUMF data* (2, 3). Usual intakes were estimated using the National Cancer Institute (NCI) method, and analyses were adjusted for age, sex, dietary misreporting status, weekend/weekday, and sequence of dietary recall. Abbreviations: d=day; g=grams; mg=milligrams; kcal=kilocalories; SE=standard error; FOPL= front-of-pack labeling; S1=scenario 1; S2=scenario 2; S3=scenario 3; S4=scenario 4. Baseline and counterfactual scenarios are described in methods. Counterfactual scenario 1 and 2 were based on Taillie, et. al. (4). **SCENARIO 1**, overall changes: sodium (mg) -4.7%; sugars (g) -10.2%; saturated fat (g) -3.9%; and **SCENARIO 2**, disaggregated by foods: sodium (mg) -4.6%; sugars (g) -5.4%; saturated fat (g) -3.6%; and beverages: sodium (mg) -5.2%; sugars (g) -13.2%; saturated fat (g) -5.6%. Counterfactual scenario 3 was based on Acton, et. al. (5). **SCENARIO 3**, relative changes disaggregated by snack foods: sodium (mg) -6.3%; sugars (g) -0.1%; saturated fat (g) -6.5%; and beverages: sodium (mg) -5.5%; sugars (g) -8.7%; saturated fat (g) -19.5%. Counterfactual scenario 4 was based on Song, et. al. (6). **SCENARIO 4**, overall changes: sodium (mg) -7.8%; sugars (g) -7.3%; saturated fat (g) -16.3%**.** * Indicates a statistically significant difference between baseline mean intakes and counterfactual mean intakes.

Table S3.5. Potential changes in percentage of total energy from saturated fat intake of implementing a 'high in' FOPL symbol in Canada (≥19 y), overall and by DRI age/sex group.

SATURATED FAT (% total energy/d)																
	**n**	**Baseline mean**	*SE*	**S1 mean**	*SE*	*S1 Δ*	**S2 mean**	*SE*	*S2 Δ*	**S3 mean**	*SE*	*S3 Δ*	**S4 mean**	*SE*	*S4 Δ*
Total 19+	***11991***	***10.56***	*0.18*	***10.39***	*0.18*	***0.17***	***10.36***	*0.18*	***0.21***	***10.29***	*0.18*	***0.27***	***9.14****	*0.16*	*1.42*
Males															
19-30 y	765	**10.73**	0.15	**10.55**	0.15	**0.18**	**10.53**	0.15	**0.20**	**10.48**	0.15	**0.25**	**9.29***	0.13	**1.44**
31-50 y	1839	**10.60**	0.15	**10.42**	0.14	**0.18**	**10.39**	0.14	**0.21**	**10.35**	0.14	**0.25**	**9.17***	0.13	**1.43**
51-70 y	1964	**10.44**	0.23	**10.27**	0.23	**0.17**	**10.23**	0.23	**0.21**	**10.19**	0.22	**0.25**	**9.03***	0.20	**1.41**
> 70 y	1105	**10.31**	0.31	**10.14**	0.31	**0.17**	**10.10**	0.31	**0.21**	**10.06**	0.30	**0.25**	**8.92***	0.27	**1.39**
Females															
19-30 y	815	**10.66**	0.15	**10.50**	0.14	**0.16**	**10.46**	0.14	**0.20**	**10.38**	0.14	**0.28**	**9.24***	0.13	**1.42**
31-50 y	2056	**10.63**	0.17	**10.46**	0.17	**0.17**	**10.42**	0.17	**0.21**	**10.34**	0.17	**0.29**	**9.21***	0.15	**1.42**
51-70 y	2107	**10.56**	0.26	**10.40**	0.25	**0.16**	**10.35**	0.25	**0.21**	**10.27**	0.25	**0.29**	**9.15***	0.22	**1.41**
> 70 y	1340	**10.43**	0.32	**10.28**	0.31	**0.15**	**10.22**	0.31	**0.21**	**10.14**	0.31	**0.29**	**9.04***	0.28	**1.39**

Baseline and counterfactual calorie and nutrient intakes were estimated using *CCHS-Nutrition 2015 PUMF data* (2, 3). Usual intakes were estimated using the National Cancer Institute (NCI) method, and analyses were adjusted for age, sex, dietary misreporting status, weekend/weekday, and sequence of dietary recall. Abbreviations: d=day; g=grams; mg=milligrams; kcal=kilocalories; SE=standard error; FOPL= front-of-pack labeling; S1=scenario 1; S2=scenario 2; S3=scenario 3; S4=scenario 4. Baseline and counterfactual scenarios are described in methods. Counterfactual scenario 1 and 2 were based on Taillie, et. al. (4). **SCENARIO 1**, overall changes: sodium (mg) -4.7%; sugars (g) -10.2%; saturated fat (g) -3.9%; and **SCENARIO 2**, disaggregated by foods: sodium (mg) -4.6%; sugars (g) -5.4%; saturated fat (g) -3.6%; and beverages: sodium (mg) -5.2%; sugars (g) -13.2%; saturated fat (g) -5.6%. Counterfactual scenario 3 was based on Acton, et. al. (5). **SCENARIO 3**, relative changes disaggregated by snack foods: sodium (mg) -6.3%; sugars (g) -0.1%; saturated fat (g) -6.5%; and beverages: sodium (mg) -5.5%; sugars (g) -8.7%; saturated fat (g) -19.5%. Counterfactual scenario 4 was based on Song, et. al. (6). **SCENARIO 4**, overall changes: sodium (mg) -7.8%; sugars (g) -7.3%; saturated fat (g) -16.3%**.** * Indicates a statistically significant difference between baseline mean intakes and counterfactual mean intakes.

Table S4. Diet related NCD deaths that could be averted or delayed in Canada due to changes in calorie and critical nutrients content of food and beverage purchases in the presence of 'high in' FOPL

Table S4.1. Scenario 1: Estimated number of deaths that could be averted or delayed in Canada after modelling reductions of critical nutrient intakes based on Chilean FOPL evaluations (overall changes: sodium -4.7%, total sugars -10.2%, sat fat -3.9%) - by cause of death (95% UI)

Cause of death (ICD-10 Code) [1]	Total (mean, 95% UI) [2]	% [3]	Men (mean, 95% UI) [2]	% [3]	Women (mean, 95% UI) [2]	% [3]
**Cardiovascular diseases**	**4403 (3916, 4892)**	**68.3**	**2419 (2153, 2683)**	**69.2**	**1982 (1682, 2262)**	**67.3**
Ischaemic heart disease (I20-25)	2271 (1996, 2548)	35.3	1481 (1302, 1656)	42.3	786 (587, 977)	26.7
Cerebrovascular disease (I60-69)	801 (614, 995)	12.4	356 (276, 432)	10.2	447 (334, 557)	15.2
Heart failure (I50)	727 (473, 969)	11.3	320 (218, 422)	9.1	402 (257, 540)	13.7
Aortic aneurysm (I71)	14 (6, 23)	0.2	9 (4, 15)	0.3	4 (2, 8)	0.1
Pulmonary embolism (I26)	3 (1, 6)	0.0	1 (0, 3)	0.0	1 (0, 3)	0.0
Rheumatic heart disease (I05-09)	2 (1, 5)	0.0	1 (0, 2)	0.0	1 (0, 3)	0.0
Hypertensive disease (I10-15)	587 (455, 705)	9.1	249 (194, 298)	7.1	337 (257, 408)	11.5
**Diabetes (E11, E14)**	**898 (684, 1088)**	**13.9**	**490 (380, 590)**	**14.0**	**409 (303, 495)**	**13.9**
**Cancer**	**631 (493, 766)**	**9.8**	**277 (207, 345)**	**7.9**	**352 (276, 430)**	**12.0**
Colorectum (C18-C20)	287 (192, 380)	4.5	150 (99, 200)	4.3	137 (92, 180)	4.7
Gallbladder (C23)	12 (8, 15)	0.2	4 (3, 5)	0.1	8 (5, 10)	0.3
Pancreas (C25)	115 (25, 205)	1.8	55 (11, 99)	1.6	57 (11, 103)	1.9
Breast (C50)	25 (-13, 61)	0.4	0	0.0	24 (-13, 62)	0.8
Endometrium (C54.1)	88 (64, 112)	1.4	0	0.0	88 (64, 111)	3.0
Kidney (C64)	106 (83, 128)	1.6	67 (53, 80)	1.9	39 (31, 47)	1.3
**Chronic renal failure (N18)**	**183 (88, 272)**	**2.8**	**93 (45, 137)**	**2.7**	**92 (40, 137)**	**3.1**
**Liver disease (K70, K74)**	**332 (200, 456)**	**5.2**	**219 (143, 292)**	**6.3**	**113 (59, 162)**	**3.8**
Total deaths prevented under 75	*2265 (2059, 2469)*	*35.2*	*1532 (1396, 1668)*	*43.8*	*733 (649, 816)*	*24.9*
**Total deaths averted or delayed**	**6442 (5870, 7020)**	**100.0**	**3498 (3193, 3802)**	**100.0**	**2943 (2618, 3259)**	**100.0**
Actual number of diet-related NCD deaths in Canada (2019)	*92845*		*46568*		*46277*	
% of actual diet-related NCD deaths that could be averted or delayed [4]	***6.9***		***7.5***		***6.4***	

1. WHO, International Statistical Classification of Diseases and Related Health Problems, Tenth Revision. 2. 95% UI are based on 10,000 iterations of Monte Carlo analysis built in PRIME. 3. Percentage from total diet-related NCD deaths that could have been prevented or delayed. 4. Percentage of actual deaths in Canada (2019) attributable to the diet-related NCDs under study. Note: total deaths averted or delayed represent less than the sum of the individual diet related NCD mortality causes given that double counting has been accounted for in PRIME during the modelling process. The same applies to the sum of CVDs and cancers.

Table S4.1.1. Sensitivity analysis (% reduction kcal reported by study). Scenario 1: Estimated number of deaths that could be averted or delayed in Canada after modelling reductions of critical nutrient intakes based on Chilean FOPL evaluations (overall changes: sodium -4.7%, calories -3.5%) - by cause of death (95% UI)

Cause of death (ICD-10 Code) [1]	Total (mean, 95% UI) [2]	% [3]	Men (mean, 95% UI) [2]	% [3]	Women (mean, 95% UI) [2]	% [3]
**Cardiovascular diseases**	**5891 (5272, 6518)**	**67.6**	**3311 (2980, 3635)**	**68.5**	**2587 (2200, 2981)**	**66.5**
Ischaemic heart disease (I20-25)	3080 (2698, 3448)	35.3	2051 (1811, 2288)	42.4	1034 (747, 1287)	26.6
Cerebrovascular disease (I60-69)	1059 (792, 1315)	12.1	480 (372, 587)	9.9	581 (431, 729)	14.9
Heart failure (I50)	970 (633, 1290)	11.1	440 (298, 572)	9.1	530 (329, 715)	13.6
Aortic aneurysm (I71)	14 (6, 23)	0.2	9 (4, 15)	0.2	5 (2, 8)	0.1
Pulmonary embolism (I26)	3 (1, 6)	0.0	2 (0, 3)	0.0	1 (0, 3)	0.0
Rheumatic heart disease (I05-09)	2 (1, 5)	0.0	1 (0, 2)	0.0	1 (0, 3)	0.0
Hypertensive disease (I10-15)	775 (601, 930)	8.9	333 (261, 400)	6.9	441 (339, 531)	11.3
**Diabetes (E11, E14)**	**1235 (934, 1491)**	**14.2**	**690 (525, 824)**	**14.3**	**546 (397, 669)**	**14.0**
**Cancer**	**892 (697, 1087)**	**10.2**	**405 (309, 505)**	**8.4**	**487 (380, 595)**	**12.5**
Colorectum (C18-C20)	408 (278, 539)	4.7	221 (150, 289)	4.6	190 (127, 249)	4.9
Gallbladder (C23)	17 (11, 22)	0.2	6 (4, 8)	0.1	10 (7, 14)	0.3
Pancreas (C25)	160 (37, 288)	1.8	83 (15, 147)	1.7	81 (17, 144)	2.1
Breast (C50)	33 (-17, 86)	0.4	0	0.0	33 (-20, 85)	0.8
Endometrium (C54.1)	121(88, 151)	1.4	0	0.0	120 (87, 152)	3.1
Kidney (C64)	151 (121, 181)	1.7	97 (78, 117)	2.0	54 (43, 64)	1.4
**Chronic renal failure (N18)**	**257 (114, 380)**	**2.9**	**131 (63, 196)**	**2.7**	**125 (57, 187)**	**3.2**
**Liver disease (K70, K74)**	**452 (266, 627)**	**5.2**	**304 (191, 409)**	**6.3**	**150 (71, 216)**	**3.9**
Total deaths prevented under 75	*3093 (2812, 3361)*	*35.5*	*2126 (1943, 2304)*	*44.0*	*969 (850, 1080)*	*24.9*
**Total deaths averted or delayed**	**8717 (7968, 9488)**	**100.0**	**4835 (4431, 5216)**	**100.0**	**3890 (3444, 4311)**	**100.0**
Actual number of diet-related NCD deaths in Canada (2019)	*92845*		*46568*		*46277*	
% of actual diet-related NCD deaths that could be averted or delayed [4]	***9.4***		***10.4***		***8.4***	

1. WHO, International Statistical Classification of Diseases and Related Health Problems, Tenth Revision. 2. 95% UI are based on 10,000 iterations of Monte Carlo analysis built in PRIME. 3. Percentage from total diet-related NCD deaths that could have been

prevented or delayed. 4. Percentage of actual deaths in Canada (2019) attributable to the diet-related NCDs under study. Note: total deaths averted or delayed represent less than the sum of the individual diet related NCD mortality causes given that double counting has been accounted for in PRIME during the modelling process. The same applies to the sum of CVDs and cancers.

Table S4.2. Scenario 2: Estimated number of deaths that could be averted or delayed in Canada after modelling reductions of critical nutrient intakes based on Chilean FOPL evaluations, disaggregated by foods and beverages (foods: sodium -4.6%, total sugars -5.4%, sat fat -3.6%; beverages: sodium -5.2%, total sugars -13.2%, sat fat -5.6%) - by cause of death (95% UI)

**Cause of death (ICD-10 Code)** [1]	**Total (mean, 95% UI)** [2]	**%** [3]	**Men (mean, 95% UI)** [2]	**%** [3]	**Women (mean, 95% UI)** [2]	**%** [3]
**Cardiovascular diseases**	**3550 (3138, 3982)**	**68.9**	**1978 (1757, 2218)**	**69.7**	**1573 (1337, 1802)**	**68.2**
Ischaemic heart disease (I20-25)	1843 (1622, 2069)	35.8	1213 (1065, 1363)	42.8	631 (476, 771)	27.3
Cerebrovascular disease (I60-69)	645 (492, 791)	12.5	291 (229, 353)	10.3	352 (264, 437)	15.3
Heart failure (I50)	576 (385, 755)	11.2	260 (176, 339)	9.2	317 (207, 422)	13.7
Aortic aneurysm (I71)	13 (6, 23)	0.3	9 (4, 15)	0.3	4 (2, 8)	0.2
Pulmonary embolism (I26)	3 (1, 6)	0.1	1 (0, 3)	0.0	1 (0, 3)	0.0
Rheumatic heart disease (I05-09)	2 (1, 5)	0.0	1 (0, 2)	0.0	1 (0, 3)	0.0
Hypertensive disease (I10-15)	473 (372, 571)	9.2	206 (162, 248)	7.3	270 (207, 326)	11.7
**Diabetes (E11, E14)**	**706 (537, 854)**	**13.7**	**391 (303, 469)**	**13.8**	**314 (232, 383)**	**13.6**
**Cancer**	**483 (377, 586)**	**9.4**	**217 (161, 270)**	**7.7**	**265 (205, 324)**	**11.5**
Colorectum (C18-C20)	221 (151, 289)	4.3	118 (79, 157)	4.2	103 (70,136)	4.5
Gallbladder (C23)	9 (6, 12)	0.2	3 (2, 4)	0.1	6 (4, 7)	0.3
Pancreas (C25)	87 (17, 156)	1.7	43 (8, 79)	1.5	43 (7, 77)	1.9
Breast (C50)	17 (-12, 45)	0.3	0	0.0	17 (-12, 46)	0.7
Endometrium (C54.1)	67 (48, 85)	1.3	0	0.0	67 (48, 85)	2.9
Kidney (C64)	82 (64, 99)	1.6	52 (42, 63)	1.8	29 (23, 36)	1.3
**Chronic renal failure (N18)**	**142 (65, 214)**	**2.8**	**72 (34, 110)**	**2.5**	**70 (31, 106)**	**3.0**
**Liver disease (K70, K74)**	**267 (164, 363)**	**5.2**	**177 (115, 239)**	**6.2**	**90 (47, 127)**	**3.9**
Total deaths prevented under 75	*1853 (1677, 2020)*	*36.0*	*1266 (1152, 1389)*	*44.6*	*583 (514, 649)*	*25.3*
**Total deaths averted or delayed**	**5151 (4672, 5610)**	**100.0**	**2836 (2583, 3100)**	**100.0**	**2308 (2049, 2568)**	**100.0**
Actual number of diet-related NCD deaths in Canada (2019)	*92845*		*46568*		*46277*	
% of actual diet-related NCD deaths that could be averted or delayed [4]	***5.5***		***6.1***		***5.0***	

1. WHO, International Statistical Classification of Diseases and Related Health Problems, Tenth Revision. 2. 95% UI are based on 10,000 iterations of Monte Carlo analysis built in PRIME. 3. Percentage from total diet-related NCD deaths that could have been prevented or delayed. 4. Percentage of actual deaths in Canada (2019) attributable to the diet-related NCDs under study. Note: total

deaths averted or delayed represent less than the sum of the individual diet related NCD mortality causes given that double counting has been accounted for in PRIME during the modelling process. The same applies to the sum of CVDs and cancers.

Table S4.2.1. Sensitivity analysis (% reduction kcal reported by study). Scenario 2: Estimated number of deaths that could be averted or delayed in Canada after modelling reductions of critical nutrient intakes based on Chilean FOPL evaluations, disaggregated by foods and beverages (foods: sodium -4.6%, calories -1.7%; beverages: sodium -5.2%, calories 9.9%) - by cause of death (95% UI)

Cause of death (ICD-10 Code) [1]	Total (mean, 95% UI) [2]	% [3]	Men (mean, 95% UI) [2]	% [3]	Women (mean, 95% UI) [2]	% [3]
**Cardiovascular diseases**	**4307 (3827, 4785)**	**68.4**	**2446 (2180, 2707)**	**69.1**	**1856 (1576, 2118)**	**67.5**
Ischaemic heart disease (I20-25)	2247 (1991, 2513)	35.7	1505 (1330, 1686)	42.5	741 (556, 914)	26.9
Cerebrovascular disease (I60-69)	777 (595, 953)	12.3	358 (279, 434)	10.1	418 (312, 521)	15.2
Heart failure (I50)	699 (456, 927)	11.1	322 (217, 424)	9.1	376 (237, 501)	13.7
Aortic aneurysm (I71)	13 (5, 23)	0.2	9 (4, 15)	0.3	4 (2, 8)	0.1
Pulmonary embolism (I26)	3 (1, 6)	0.0	1 (0, 3)	0.0	1 (0, 3)	0.0
Rheumatic heart disease (I05-09)	2 (1, 5)	0.0	1 (0, 2)	0.0	1 (0, 3)	0.0
Hypertensive disease (I10-15)	567 (438, 685)	9.0	250 (194, 301)	7.1	316 (242, 384)	11.5
**Diabetes (E11, E14)**	**878 (670, 1052)**	**14.0**	**498 (385, 597)**	**14.1**	**381 (279, 465)**	**13.8**
**Cancer**	**608 (472, 739)**	**9.7**	**281 (212, 349)**	**7.9**	**325 (252, 398)**	**11.8**
Colorectum (C18-C20)	281 (188, 369)	4.5	153 (103, 201)	4.3	127 (85, 166)	4.6
Gallbladder (C23)	11 (8, 15)	0.2	4 (3, 6)	0.1	7 (5, 9)	0.3
Pancreas (C25)	110 (23, 196)	1.7	56 (11, 103)	1.6	53 (10, 94)	1.9
Breast (C50)	20 (-14, 56)	0.3	0	0.0	20 (-15, 56)	0.7
Endometrium (C54.1)	82 (60, 104)	1.3	0	0.0	82 (59, 104)	3.0
Kidney (C64)	104 (82, 125)	1.7	68 (54, 82)	1.9	36 (29, 43)	1.3
**Chronic renal failure (N18)**	**178 (85, 268)**	**2.8**	**93 (46, 140)**	**2.6**	**85 (39, 131)**	**3.1**
**Liver disease (K70, K74)**	**329 (206, 453)**	**5.2**	**223 (141, 299)**	**6.3**	**107 (56, 153)**	**3.9**
Total deaths prevented under 75	*2274 (2080, 2469)*	*36.1*	*1578 (1433, 1716)*	*44.6*	*697 (614, 775)*	*25.3*
**Total deaths averted or delayed**	**6293 (5753, 6835)**	**100.0**	**3540 (3230, 3842)**	**100.0**	**2751 (2443, 3040)**	**100.0**
Actual number of diet-related NCD deaths in Canada (2019)	*92845*		*46568*		*46277*	
% of actual diet-related NCD deaths that could be averted or delayed [4]	***6.8***		***7.6***		***5.9***	

1. WHO, International Statistical Classification of Diseases and Related Health Problems, Tenth Revision. 2. 95% UI are based on 10,000 iterations of Monte Carlo analysis built in PRIME. 3. Percentage from total diet-related NCD deaths that could have been

prevented or delayed. 4. Percentage of actual deaths in Canada (2019) attributable to the diet-related NCDs under study. Note: total deaths averted or delayed represent less than the sum of the individual diet related NCD mortality causes given that double counting has been accounted for in PRIME during the modelling process. The same applies to the sum of CVDs and cancers.

Table S4.3. Scenario 3: Estimated number of deaths that could be averted or delayed in Canada after modelling reductions on snack foods and beverages based on a Canadian randomized experimental marketplace study (snack foods: sodium -6.3%, total sugars -0.1%, sat fat -6.5%; beverages: sodium -5.5%, total sugars -8.7%, sat fat -19.5%) - by cause of death (95% UI)

Cause of death (ICD-10 Code) [1]	Total (mean, 95% UI) [2]	% [3]	Men (mean, 95% UI) [2]	% [3]	Women (mean, 95% UI) [2]	% [3]
**Cardiovascular diseases**	**1495 (1346, 1640)**	**68.5**	**858 (776, 940)**	**68.9**	**636 (547, 723)**	**67.7**
Ischaemic heart disease (I20-25)	817 (731, 901)	37.4	545 (486, 604)	43.8	272 (214, 327)	29.0
Cerebrovascular disease (I60-69)	251 (193, 308)	11.5	117 (91, 142)	9.4	134 (99, 167)	14.3
Heart failure (I50)	235 (154, 316)	10.8	110 (74, 145)	8.8	125 (82, 167)	13.3
Aortic aneurysm (I71)	3 (1, 5)	0.1	2 (1, 3)	0.2	1 (0, 2)	0.1
Pulmonary embolism (I26)	1 (0, 1)	0.0	0 (0, 1)	0.0	0 (0, 1)	0.0
Rheumatic heart disease (I05-09)	1 (0, 1)	0.0	0 (0, 0)	0.0	0 (0, 1)	0.0
Hypertensive disease (I10-15)	188 (145, 230)	8.6	84 (66, 102)	6.7	104 (80, 128)	11.1
**Diabetes (E11, E14)**	**308 (237, 372)**	**14.1**	**177 (139, 212)**	**14.2**	**131 (97, 159)**	**14.0**
**Cancer**	**200 (156, 246)**	**9.2**	**95 (72, 119)**	**7.6**	**105 (82, 129)**	**11.2**
Colorectum (C18-C20)	93 (64, 123)	4.3	52 (35, 68)	4.2	41 (28, 54)	4.4
Gallbladder (C23)	4 (2, 5)	0.2	1 (1, 2)	0.1	2 (1, 3)	0.2
Pancreas (C25)	36 (7, 65)	1.6	19 (3, 34)	1.5	17 (3, 31)	1.8
Breast (C50)	5 (-6, 17)	0.2	0	0.0	5 (-6, 17)	0.5
Endometrium (C54.1)	28 (20, 35)	1.3	0	0.0	27 (20, 35)	2.9
Kidney (C64)	35 (28, 42)	1.6	23 (18, 28)	1.8	12 (9, 14)	1.3
**Chronic renal failure (N18)**	**60 (29, 90)**	**2.7**	**32 (15, 49)**	**2.6**	**28 (12, 43)**	**3.0**
**Liver disease (K70, K74)**	**121 (75, 166)**	**5.5**	**83 (54, 111)**	**6.7**	**39 (22, 55)**	**4.2**
Total deaths prevented under 75	*824 (757, 890)*	*37.7*	*574 (529, 618)*	*46.1*	*251 (224, 277)*	*26.7*
**Total deaths averted or delayed**	**2183 (2008, 2361)**	**100.0**	**1245 (1148, 1340)**	**100.0**	**939 (843, 1035)**	**100.0**
Actual number of diet-related NCD deaths in Canada (2019)	*92845*		*46568*		*46277*	
% of actual diet-related NCD deaths that could be averted or delayed [4]	***2.4***		***2.7***		***2.0***	

1. WHO, International Statistical Classification of Diseases and Related Health Problems, Tenth Revision. 2. 95% UI are based on 10,000 iterations of Monte Carlo analysis built in PRIME. 3. Percentage from total diet-related NCD deaths that could have been prevented or delayed. 4. Percentage of actual deaths in Canada (2019) attributable to the diet-related NCDs under study. Note: total

deaths averted or delayed represent less than the sum of the individual diet related NCD mortality causes given that double counting has been accounted for in PRIME during the modelling process. The same applies to the sum of CVDs and cancers.

Table S4.3.1. Sensitivity analysis (% reduction kcal reported by study). Scenario 3: Estimated number of deaths that could be averted or delayed in Canada after modelling reductions on snack foods and beverages based on a Canadian randomized experimental marketplace study (snack foods: sodium -6.3%, calories -3.0%; beverages: sodium -5.5%, calories -10.5%) - by cause of death (95% UI)

**Cause of death (ICD-10 Code)** [1]	**Total (mean, 95% UI)** [2]	**%** [3]	**Men (mean, 95% UI)** [2]	**%** [3]	**Women (mean, 95% UI)** [2]	**%** [3]
**Cardiovascular diseases**	**2843 (2552, 3121)**	**67.0**	**1543 (1402, 1691)**	**68.9**	**1296 (1116, 1476)**	**66.2**
Ischaemic heart disease (I20-25)	1497 (1326, 1665)	35.3	966 (855, 1073)	43.8	527 (397, 657)	26.9
Cerebrovascular disease (I60-69)	501 (380, 619)	11.8	217 (167, 266)	9.4	284 (211, 357)	14.5
Heart failure (I50)	472 (309, 634)	11.1	207 (134, 275)	8.8	267 (166, 357)	13.6
Aortic aneurysm (I71)	3 (1, 5)	0.1	2 (1, 3)	0.2	1 (0, 2)	0.1
Pulmonary embolism (I26)	1 (0, 1)	0.0	0 (0, 1)	0.0	0 (0, 1)	0.0
Rheumatic heart disease (I05-09)	1 (0, 1)	0.0	0 (0, 0)	0.0	0 (0, 1)	0.0
Hypertensive disease (I10-15)	369 (283, 448)	8.7	153 (120, 186)	6.7	217 (164, 264)	11.1
**Diabetes (E11, E14)**	**622 (471, 756)**	**14.7**	**337 (262, 407)**	**14.2**	**283 (212, 343)**	**14.5**
**Cancer**	**421 (328, 514)**	**9.9**	**185 (140, 232)**	**7.6**	**237 (184, 288)**	**12.1**
Colorectum (C18-C20)	192 (129, 253)	4.5	101 (68, 134)	4.2	92 (62, 122)	4.7
Gallbladder (C23)	8 (5, 10)	0.2	3 (2, 4)	0.1	5 (3, 7)	0.3
Pancreas (C25)	77 (15, 138)	1.8	37 (8, 67)	1.5	39 (8, 69)	2.0
Breast (C50)	14 (-11, 39)	0.3	0	0.0	15 (-10, 40)	0.8
Endometrium (C54.1)	60 (43, 76)	1.4	0	0.0	60 (43, 76)	3.1
Kidney (C64)	71 (57, 86)	1.7	45 (36, 54)	1.8	26 (21, 32)	1.3
**Chronic renal failure (N18)**	**125 (57, 187)**	**2.9**	**62 (29, 94)**	**2.6**	**62 (29, 94)**	**3.2**
**Liver disease (K70, K74)**	**234 (144, 323)**	**5.5**	**155 (99, 206)**	**6.7**	**81 (45, 114)**	**4.1**
Total deaths prevented under 75	*1528 (1403, 1651)*	*36.0*	*1027 (945, 1110)*	*45.0*	*501 (446, 556)*	*25.6*
**Total deaths averted or delayed**	**4243 (3883, 4583)**	**100.0**	**2283 (2104, 2466)**	**100.0**	**1957 (1757, 2163)**	**100.0**
Actual number of diet-related NCD deaths in Canada (2019)	*92845*		*46568*		*46277*	
% of actual diet-related NCD deaths that could be averted or delayed [4]	***4.6***		***4.9***		***4.2***	

1. WHO, International Statistical Classification of Diseases and Related Health Problems, Tenth Revision. 2. 95% UI are based on 10,000 iterations of Monte Carlo analysis built in PRIME. 3. Percentage from total diet-related NCD deaths that could have been

prevented or delayed. 4. Percentage of actual deaths in Canada (2019) attributable to the diet-related NCDs under study. Note: total deaths averted or delayed represent less than the sum of the individual diet related NCD mortality causes given that double counting has been accounted for in PRIME during the modelling process. The same applies to the sum of CVDs and cancers.

Table S4.4. Scenario 4: Estimated number of deaths that could be averted or delayed after modelling reductions of targeted nutrient intakes based on a meta-analysis that looked at the impact of warning labels on changing consumers' food and beverages purchasing behavior (overall changes sodium -7.8%, total sugars -7.3%, sat fat -16.3%) - by cause of death (95% UI)

Cause of death (ICD-10 Code) [1]	Total (mean, 95% UI) [2]	% [3]	Men (mean, 95% UI) [2]	% [3]	Women (mean, 95% UI) [2]	% [3]
**Cardiovascular diseases**	**6253 (5555, 6912)**	**70.2**	**3528 (3150, 3903)**	**71.3**	**2716 (2319, 3093)**	**68.8**
Ischaemic heart disease (I20-25)	3456 (3068, 3839)	38.8	2277 (2023, 2521)	46.0	1180 (918, 1422)	29.9
Cerebrovascular disease (I60-69)	1035 (783, 1282)	11.6	471 (359, 580)	9.5	563 (414, 709)	14.3
Heart failure (I50)	950 (630, 1245)	10.7	429 (288, 558)	8.7	520 (333, 684)	13.2
Aortic aneurysm (I71)	23 (10, 38)	0.3	15 (6, 26)	0.3	7 (3, 12)	0.2
Pulmonary embolism (I26)	5 (1, 10)	0.1	3 (1, 5)	0.1	2 (1, 5)	0.1
Rheumatic heart disease (I05-09)	4 (1, 8)	0.0	2 (0, 4)	0.0	2 (1, 5)	0.1
Hypertensive disease (I10-15)	782 (601, 936)	8.8	336 (264, 402)	6.8	442 (341, 533)	11.2
**Diabetes (E11, E14)**	**1157 (877, 1392)**	**13.0**	**642 (495, 769)**	**13.0**	**517 (373, 631)**	**13.1**
**Cancer**	**834 (644, 1017)**	**9.4**	**375 (283, 465)**	**7.6**	**458 (355, 561)**	**11.6**
Colorectum (C18-C20)	384 (259, 506)	4.3	203 (138, 268)	4.1	179 (120, 235)	4.5
Gallbladder (C23)	15 (10, 20)	0.2	6 (4, 7)	0.1	10 (7, 13)	0.3
Pancreas (C25)	150 (29, 268)	1.7	76 (16, 135)	1.5	76 (13, 134)	1.9
Breast (C50)	31 (-18, 80)	0.3	0	0.0	31 (-19, 79)	0.8
Endometrium (C54.1)	114 (83, 143)	1.3	0	0.0	114 (82, 144)	2.9
Kidney (C64)	140 (112, 169)	1.6	90 (72, 108)	1.8	51 (40, 61)	1.3
**Chronic renal failure (N18)**	**242 (110, 358)**	**2.7**	**121 (55, 180)**	**2.4**	**118 (53, 177)**	**3.0**
**Liver disease (K70, K74)**	**427 (253, 585)**	**4.8**	**285 (181, 381)**	**5.8**	**143 (70, 206)**	**3.6**
Total deaths prevented under 75	*3238 (2952, 3518)*	*36.4*	*2237 (2039, 2427)*	*45.2*	*1004 (891, 1113)*	*25.4*
**Total deaths averted or delayed**	**8907 (8095, 9667)**	**100.0**	**4949 (4521, 5363)**	**100.0**	**3947 (3513, 4366)**	**100.0**
Actual number of diet-related NCD deaths in Canada (2019)	*92845*		*46568*		*46277*	
% of actual diet-related NCD deaths that could be averted or delayed [4]	***9.6***		***10.6***		***8.5***	

1. WHO, International Statistical Classification of Diseases and Related Health Problems, Tenth Revision. 2. 95% UI are based on 10,000 iterations of Monte Carlo analysis built in PRIME. 3. Percentage from total diet-related NCD deaths that could have been prevented or delayed. 4. Percentage of actual deaths in Canada (2019) attributable to the diet-related NCDs under study. Note: total

deaths averted or delayed represent less than the sum of the individual diet related NCD mortality causes given that double counting has been accounted for in PRIME during the modelling process. The same applies to the sum of CVDs and cancers.

Table S4.4.1. Sensitivity analysis (% reduction kcal reported by study). Scenario 4: Estimated number of deaths that could be averted or delayed after modelling reductions of targeted nutrient intakes based on a meta-analysis that looked at the impact of warning labels on changing consumers' food and beverages purchasing behavior (overall changes: sodium -7.8%, calories -12.9%) - by cause of death (95% UI)

Cause of death (ICD-10 Code) [1]	Total (mean, 95% UI) [2]	% [3]	Men (mean, 95% UI) [2]	% [3]	Women (mean, 95% UI) [2]	% [3]
**Cardiovascular diseases**	**15088 (13088, 16926)**	**67.3**	**8908 (7948, 9805)**	**69.4**	**6138 (4681, 7448)**	**64.2**
Ischaemic heart disease (I20-25)	8715 (7419, 9933)	38.9	6045 (5341, 6711)	47.1	2671 (1486, 3663)	27.9
Cerebrovascular disease (I60-69)	2306 (1333, 3163)	10.3	1048 (643, 1401)	8.2	1241 (686, 1758)	13.0
Heart failure (I50)	2239 (1106, 3119)	10.0	1018 (520, 1389)	7.9	1218 (537, 1692)	12.7
Aortic aneurysm (I71)	23 (9, 39)	0.1	15 (6, 26)	0.1	7 (3, 12)	0.1
Pulmonary embolism (I26)	5 (2, 10)	0.0	2 (1, 5)	0.0	2 (1, 5)	0.0
Rheumatic heart disease (I05-09)	4 (1, 8)	0.0	2 (0, 3)	0.0	2 (1, 5)	0.0
Hypertensive disease (I10-15)	1858 (1278, 2239)	8.3	802 (562, 960)	6.2	1049 (701, 1279)	11.0
**Diabetes (E11, E14)**	**2926 (1675, 3710)**	**13.1**	**1654 (977, 2064)**	**12.9**	**1289 (676, 1649)**	**13.5**
**Cancer**	**2899 (2252, 3499)**	**12.9**	**1363 (1045, 1669)**	**10.6**	**1546 (1214, 1877)**	**16.2**
Colorectum (C18-C20)	1364 (924, 1771)	6.1	747 (515, 965)	5.8	622 (435, 808)	6.5
Gallbladder (C23)	53 (37, 69)	0.2	20 (14, 26)	0.2	34 (24, 43)	0.4
Pancreas (C25)	550 (120, 948)	2.5	282 (64, 488)	2.2	269 (59, 464)	2.8
Breast (C50)	99 (-80, 273)	0.4	0	0.0	101 (-77, 272)	1.1
Endometrium (C54.1)	360 (272, 434)	1.6	0	0.0	359 (273, 433)	3.8
Kidney (C64)	483 (393, 569)	2.2	315 (256, 371)	2.5	168 (137, 197)	1.8
**Chronic renal failure (N18)**	**685 (191, 1037)**	**3.1**	**357 (100, 523)**	**2.8**	**334 (76, 500)**	**3.5**
**Liver disease (K70, K74)**	**906 (166, 1456)**	**4.0**	**620 (160, 957)**	**4.8**	**289 (-3, 501)**	**3.0**
Total deaths prevented under 75	*8114 (7213, 8885)*	*36.2*	*5695 (5127, 6202)*	*44.4*	*2412 (1980, 2782)*	*25.2*
**Total deaths averted or delayed**	**22412 (19912, 24707)**	**100.0**	**12835 (11567, 13995)**	**100.0**	**9558 (7871, 11020)**	**100.0**
Actual number of diet-related NCD deaths in Canada (2019)	*92845*		*46568*		*46277*	
% of actual diet-related NCD deaths that could be averted or delayed [4]	***24.1***		***27.6***		***20.7***	

1. WHO, International Statistical Classification of Diseases and Related Health Problems, Tenth Revision. 2. 95% UI are based on 10,000 iterations of Monte Carlo analysis built in PRIME. 3. Percentage from total diet-related NCD deaths that could have been

prevented or delayed. 4. Percentage of actual deaths in Canada (2019) attributable to the diet-related NCDs under study. Note: total deaths averted or delayed represent less than the sum of the individual diet related NCD mortality causes given that double counting has been accounted for in PRIME during the modelling process. The same applies to the sum of CVDs and cancers.

Table S5. Sensitivity analysis using the criteria that WHO used to estimate FOPL cost-effectiveness (7)

Baseline and WHO criteria scenario (calorie, sodium, and saturated fat; ≥19 y)												
	Energy (kcal/d)	*SE*	***WHO criteria***	*SE*	***Sodium (mg/d)***	*SE*	***WHO criteria***	*SE*	***Sat fats (g/d)***	*SE*	***WHO criteria***	*SE*
Total 19+	***1889***	*20*	***1793****	*20*	***2729***	*33*	***2555****	*31*	***22.84***	*0.56*	***19.90****	***0.49***
Males												
19-30 y	**2373**	51	**2254**	48	**3435**	85	**3216**	80	**28.87**	0.82	**25.16***	0.71
31-50 y	**2236**	36	**2124**	34	**3247**	66	**3040**	61	**27.01**	0.71	**23.53***	0.62
51-70 y	**2039**	54	**1937**	52	**2974**	86	**2784**	81	**24.39**	1.13	**21.25**	0.99
> 70 y	**1905**	88	**1810**	84	**2775**	126	**2598**	118	**22.56**	1.60	**19.65**	1.40
Females												
19-30 y	**1710**	78	**1623**	74	**88**	122	**2292**	114	**20.83**	1.00	**18.15**	0.87
31-50 y	**1665**	32	**1580**	30	**85**	52	**2235**	48	**20.28**	0.38	**17.67***	0.33
51-70 y	**1585**	25	**1505**	24	**81**	31	**2127***	29	**19.30**	0.61	**16.81***	0.53
> 70 y	**1464**	36	**1390**	34	**74**	45	**1973**	42	**17.74**	0.80	**15.45**	0.70

Baseline and counterfactual calorie and nutrient intakes were estimated using *CCHS-Nutrition 2015 PUMF data* (2, 3). Usual intakes were estimated using the National Cancer Institute (NCI) method, and analyses were adjusted for age, sex, dietary misreporting status, weekend/weekday, and sequence of dietary recall. Abbreviations: d=day; g=grams; mg=milligrams; kcal=kilocalories; SE=standard error; FOPL= front-of-pack labeling; S1=scenario 1; S2=scenario 2; S3=scenario 3; S4=scenario 4. Sensitivity analysis was based on the criteria that WHO (7) used to estimate FOPL cost-effectiveness. Overall changes: sodium (mg) -6.4%; saturated fat (g) -12.9%; calories (kcal) 5.3%. * Indicates a statistically significant difference between baseline mean intakes and “counterfactual” mean intakes.

Table S5.1. Sensitivity analysis: Estimated number of deaths that could be averted or delayed after modelling reductions of targeted nutrient intakes based on WHO criteria used to estimate FOPL cost-effectiveness (overall changes sodium -6.4%, sat fat %TE -6.8%, calories -5.3%) - by cause of death (95% UI)

Cause of death (ICD-10 Code) [1]	Total (mean, 95% UI) [2]	% [3]	Men (mean, 95% UI) [2]	% [3]	Women (mean, 95% UI) [2]	% [3]
**Cardiovascular diseases**	**8427 (7520, 9320)**	**67.9**	**4805 (4328, 5265)**	**69.0**	**3642 (3063, 4188)**	**66.4**
Ischaemic heart disease (I20-25)	4562 (4006, 5093)	36.7	3061 (2725, 3403)	44.0	1512 (1077, 1902)	27.6
Cerebrovascular disease (I60-69)	1450 (1070, 1835)	11.7	661 (499, 814)	9.5	795 (566, 1014)	14.5
Heart failure (I50)	1337 (831, 1782)	10.8	604 (392, 798)	8.7	733 (440, 975)	13.4
Aortic aneurysm (I71)	19 (7, 31)	0.2	13 (5, 21)	0.2	6 (3, 10)	0.1
Pulmonary embolism (I26)	4 (1, 8)	0.0	2 (1, 4)	0.0	2 (1, 4)	0.0
Rheumatic heart disease (I05-09)	3 (1, 7)	0.0	1 (0, 3)	0.0	2 (1, 4)	0.0
Hypertensive disease (I10-15)	1069 (818, 1282)	8.6	464 (364, 553)	6.7	609 (454, 733)	11.1
**Diabetes (E11, E14)**	**1715 (1230, 2079)**	**13.8**	**956 (718, 1156)**	**13.7**	**756 (528, 923)**	**13.8**
**Cancer**	**1322 (1036, 1612)**	**10.6**	**603 (458, 745)**	**8.7**	**717 (559, 877)**	**13.1**
Colorectum (C18-C20)	613 (415, 800)	4.9	330 (221, 429)	4.7	280 (193, 368)	5.1
Gallbladder (C23)	24 (16, 32)	0.2	9 (6, 12)	0.1	15 (10, 20)	0.3
Pancreas (C25)	240 (50, 427)	1.9	122 (22, 216)	1.8	120 (23, 212)	2.2
Breast (C50)	48 (-31, 125)	0.4	0	0.0	48 (-32, 125)	0.9
Endometrium (C54.1)	176 (129, 217)	1.4	0	0.0	175 (128, 220)	3.2
Kidney (C64)	222 (178, 266)	1.8	144 (115, 171)	2.1	79 (63, 94)	1.4
**Chronic renal failure (N18)**	**365 (161, 539)**	**2.9**	**187 (88, 275)**	**2.7**	**179 (71, 264)**	**3.3**
**Liver disease (K70, K74)**	**619 (333, 868)**	**5.0**	**417 (245, 571)**	**6.0**	**203 (86, 296)**	**3.7**
Total deaths prevented under 75	*4460 (4064, 4833)*	*35.9*	*3090 (2835, 3337)*	*44.4*	*1374 (1202, 1540)*	*25.0*
**Total deaths averted or delayed**	**12416 (11351, 13469)**	**100.0**	**6960 (6383, 7512)**	**100**	**5486 (4839, 6095)**	**100**
Actual number of diet-related NCD deaths in Canada (2019)	*92845*		*46568*		*46277*	
% of actual diet-related NCD deaths that could be averted or delayed [4]	***13.4***		***14.9***		***11.9***	

1. WHO, International Statistical Classification of Diseases and Related Health Problems, Tenth Revision. 2. 95% UI are based on 10,000 iterations of Monte Carlo analysis built in PRIME. 3. Percentage from total diet-related NCD deaths that could have been prevented or delayed. 4. Percentage of actual deaths in Canada (2019) attributable to the diet-related NCDs under study. Note: total

deaths averted or delayed represent less than the sum of the individual diet related NCD mortality causes given that double counting has been accounted for in PRIME during the modelling process. The same applies to the sum of CVDs and cancers.

Table S6. Age- and sex-specific estimates of the annual number of diet related NCD deaths in Canada, 2019 (8-12)

**Males**	**20-24**	**25-29**	**30-34**	**35-39**	**40-44**	**45-49**	**50-54**	**55-59**	**60-64**	**65-69**	**70-74**	**75-79**	**80-84**	**85+**
I60-I69: Cerebrovascular diseases	3	7	14	22	30	72	101	190	280	452	641	829	1,018	2,288
I20-I25: Ischaemic heart diseases	2	8	17	65	122	269	573	1,049	1,631	1,999	2,324	2,394	2,691	5,626
C00-C14: Lip, oral cavity and pharynx	2	2	3	4	11	28	53	110	158	158	185	131	86	126
C15: Oesophagus	0	0	3	9	17	33	76	158	238	281	273	232	203	198
C16: Stomach	0	4	5	7	12	22	48	91	130	162	175	175	172	241
C34: Bronchus and lung	2	3	4	12	26	57	211	589	1,133	1,590	1,946	1,815	1,440	1,551
C25: Pancreas	0	3	4	3	13	39	92	200	334	374	475	429	321	431
C18-20: Colorectum	3	5	22	34	61	104	172	318	440	564	680	613	703	1,031
C50: Breast	-	-	-	-	-	-	-	-	-	-	-	-	-	-
C54.1: Endometrium	-	-	-	-	-	-	-	-	-	-	-	-	-	-
C23: Gallbladder	0	0	0	0	1	3	4	6	5	18	15	14	9	19
C64: Kidney	0	0	1	6	8	14	49	89	129	163	191	151	146	225
I10-I15: Hypertensive disease	0	0	5	6	14	27	58	97	117	136	156	167	244	620
E11, E14: Diabetes	5	4	13	16	42	64	100	225	294	390	538	526	522	923
C67: Bladder cancer	0	0	1	2	2	11	16	39	90	136	197	215	289	545
C22: Liver cancer	1	3	3	10	15	15	67	180	291	367	364	308	246	229
C53: Cervix cancer	-	-	-	-	-	-	-	-	-	-	-	-	-	-
K70, K74: Liver disease	0	13	17	35	62	115	188	329	363	417	307	229	172	117
I50: Heart failure	0	2	1	0	9	4	35	47	95	119	232	293	480	1,534
I71: Aortic aneurysm	1	1	6	11	12	22	32	41	79	90	141	104	144	260
I26: Pulmonary embolism	2	3	2	1	4	10	15	27	22	25	47	43	31	59
I05-09: Rheumatic heart disease	0	0	1	2	4	3	5	6	10	9	29	34	35	75
N18: Chronic renal failure	1	1	0	2	5	8	7	24	42	72	97	152	183	508

**Females**	**20-24**	**25-29**	**30-34**	**35-39**	**40-44**	**45-49**	**50-54**	**55-59**	**60-64**	**65-69**	**70-74**	**75-79**	**80-84**	**85+**
I60-I69: Cerebrovascular diseases	3	7	16	19	26	56	89	138	221	322	516	746	1,044	4,494
I20-I25: Ischaemic heart diseases	1	1	3	14	32	70	142	263	511	717	1,067	1,216	1,845	7,230
C00-C14: Lip, oral cavity, and pharynx	1	0	1	2	4	12	16	25	47	43	59	63	50	91
C15: Oesophagus	0	0	1	1	3	6	13	28	53	64	75	82	56	99
C16: Stomach	0	2	5	5	15	14	29	50	49	80	94	106	112	184
C34: Bronchus and lung	0	1	4	12	36	74	187	612	1,009	1,326	1,690	1,596	1,242	1,523
C25: Pancreas	0	1	4	5	12	32	68	154	221	298	374	376	385	565
C18-20: Colorectum	1	1	10	22	56	69	136	192	266	334	430	501	549	1,285
C50: Breast	1	2	42	62	119	193	350	454	508	570	624	585	579	1,179
C54.1: Endometrium	1	0	1	2	9	17	24	66	117	139	164	119	117	128
C23: Gallbladder	0	0	0	0	1	0	7	5	10	17	24	27	24	38
C64: Kidney	0	0	1	1	3	8	16	42	41	63	75	79	91	189
I10-I15: Hypertensive disease	0	0	0	2	7	14	15	27	61	92	124	166	259	1,418
E11, E14: Diabetes	2	3	7	14	14	25	56	112	155	249	308	342	428	1,157
C67: Bladder cancer	0	0	0	1	5	7	11	33	21	52	67	95	99	281
C22: Liver cancer	1	1	0	5	13	15	35	75	117	142	160	152	180	237
C53: Cervix cancer	2	3	12	25	37	36	42	60	46	44	38	29	20	39
K70, K74: Liver disease	2	6	17	28	32	65	104	157	172	203	156	141	95	117
I50: Heart failure	0	2	1	2	3	4	6	26	43	77	159	243	415	2,465
I71: Aortic aneurysm	0	0	1	1	2	3	7	7	18	38	60	84	109	284
I26: Pulmonary embolism	3	2	1	6	8	6	11	18	22	36	45	44	40	115
I05-09: Rheumatic heart disease	1	1	1	2	3	3	6	9	12	29	31	51	63	190
N18: Chronic renal failure	0	4	3	2	1	2	15	14	25	49	79	114	156	552

Table S7. Age- and sex-specific estimates of the Canadian population, 2019 (13)

Age	Male	Female
**20-24**	1,292,739	1,182,807
**25-29**	1,353,893	1,272,165
**30-34**	1,319,176	1,285,925
**35-39**	1,288,783	1,292,390
**40-44**	1,198,765	1,223,278
**45-49**	1,190,901	1,206,950
**50-54**	1,246,625	1,257,812
**55-59**	1,368,185	1,383,192
**60-64**	1,236,748	1,276,958
**65-69**	1,017,348	1,080,436
**70-74**	817,561	890,019
**75-79**	543,317	621,481
**80-84**	348,047	440,404
**85+**	304,616	531,841

References

1. Health Canada. Table of Reference Amounts for Food (2016) [cited 2019 July 15]. Available from: https://www.canada.ca/en/health-canada/services/technical-documents-labelling-requirements/table-reference-amounts-food.html.
2. Statistics Canada. Canadian Community Health Survey – Nutrition: Public Use Microdata File (2019) [cited 2019 October 12]. Available from: https://www150.statcan.gc.ca/n1/en/catalogue/82M0024X.
3. Health Canada. 2015 Canadian Community Health Survey - Nutrition. Reference Guide to Understanding and Using the Data (2017) [cited 2022 October 12]. Available from: https://www.canada.ca/en/health-canada/services/food-nutrition/food-nutrition-surveillance/health-nutrition-surveys/canadian-community-health-survey-cchs/reference-guide-understanding-using-data-2015.html.
4. Taillie LS, Bercholz M, Popkin B, Reyes M, Colchero MA, Corvalán C. Changes in Food Purchases after the Chilean Policies on Food Labelling, Marketing, and Sales in Schools: A before and after Study. *The Lancet Planetary Health* (2021) 5(8):e526-e33.
5. Acton RB, Jones AC, Kirkpatrick SI, Roberto CA, Hammond D. Taxes and Front-of-Package Labels Improve the Healthiness of Beverage and Snack Purchases: A Randomized Experimental Marketplace. *International Journal of Behavioral Nutrition and Physical Activity* (2019) 16(1):1-15.
6. Song J, Brown MK, Tan M, MacGregor GA, Webster J, Campbell NR, et al. Impact of Color-Coded and Warning Nutrition Labelling Schemes: A Systematic Review and Network Meta-Analysis. *PLoS Medicine* (2021) 18(10):e1003765.
7. World Health Organization. Technical Briefing for Appendix 3 of the Global Action Plan for Non-Communicable Diseases (2022) [cited 2022 October 12]. Available from: https://www.who.int/teams/noncommunicable-diseases/updating-appendix-3-of-the-who-global-ncd-action-plan-2013-2030.
8. Statistics Canada. Table 13-10-0142-01 Deaths, by Cause, Chapter Ii: Neoplasms (C00 to D48) (2019) [cited 2021 January 4]. Available from: https://www150.statcan.gc.ca/t1/tbl1/en/tv.action?pid=1310014201.
9. Statistics Canada. Table 13-10-0144-01 Deaths, by Cause, Chapter Iv: Endocrine, Nutritional and Metabolic Diseases (E00 to E90) (2019) [cited 2021 January 4]. Available from: https://www150.statcan.gc.ca/t1/tbl1/en/tv.action?pid=1310014401.
10. Statistics Canada. Table 13-10-0147-01 Deaths, by Cause, Chapter Ix: Diseases of the Circulatory System (I00 to I99) (2019) [cited 2021 January 4]. Available from: https://www150.statcan.gc.ca/t1/tbl1/en/tv.action?pid=1310014701.
11. Statistics Canada. Table 13-10-0151-01 Deaths, by Cause, Chapter Xiv: Diseases of the Genitourinary System (N00 to N99) (2019) [cited 2021 January 4]. Available from: https://www150.statcan.gc.ca/t1/tbl1/en/tv.action?pid=1310015101.
12. Statistics Canada. Table 13-10-0148-01 Deaths, by Cause, Chapter Xi: Diseases of the Digestive System (K00 to K93) (2019) [cited 2021 January 4]. Available from: https://www150.statcan.gc.ca/t1/tbl1/en/tv.action?pid=1310014801.
13. Statistics Canada. Table 17-10-0005-01 Population Estimates on July 1st, by Age and Sex (2019) [cited 2021 January 4]. Available from: https://www150.statcan.gc.ca/t1/tbl1/en/tv.action?pid=1710000501.
